# Strategies Used for Implementing and Promoting Adherence to Antibiotic Guidelines in Low- and Lower-Middle-Income Countries: A Systematic Review

**DOI:** 10.3390/tropicalmed6030166

**Published:** 2021-09-13

**Authors:** Nicola D. Foxlee, Nicola Townell, Claire Heney, Lachlan McIver, Colleen L. Lau

**Affiliations:** 1 Department of Global Health, Research School of Population Health, Australian National University, Canberra, ACT 2600, Australia; colleen.lau@uq.edu.au; 2 Infectious Disease Department, Sunshine Coast University Hospital, Birtinya, QLD 4575, Australia; nikkitownell@hotmail.com; 3 Pathology Queensland, Central Microbiology, Brisbane, QLD 4006, Australia; claire.heney@health.qld.gov.au; 4 Rocketship Pacific Ltd., Port Melbourne, Melbourne, VIC 3207, Australia; lachlan@rocket-ship.org; 5 School of Public Health, University of Queensland, Herston, QLD 4006, Australia

**Keywords:** antimicrobial resistance, antimicrobial stewardship, antibiotics, clinical guidelines, low- and lower-middle-income countries, prescribing practices

## Abstract

Containing antimicrobial resistance and reducing high levels of antibiotic consumption in low- and lower middle-income countries are a major challenge. Clinical guidelines targeting antibiotic prescribing can reduce consumption, however, the degrees to which clinical guidelines are adopted and adhered to are challenging for developers, policy makers and users. The aim of this study was to review the strategies used for implementing and promoting antibiotic guideline adherence in low- and lower middle-income countries. A review of published literature was conducted using PubMed, Cochrane Library, SCOPUS and the information systems of the World Health Organization and the Australian National University according to PRISMA guidelines and our PROSPERO protocol. The strategies were grouped into five broad categories based on the Cochrane Effective Practice and Organization of Care taxonomy. The 33 selected studies, representing 16 countries varied widely in design, setting, disease focus, methods, intervention components, outcomes and effects. The majority of interventions were multifaceted and resulted in a positive direction of effect. The nature of the interventions and study variability made it impossible to tease out which strategies had the greatest impact on improving CG compliance. Audit and feedback coupled with either workshops and/or focus group discussions were the most frequently used intervention components. All the reported strategies are established practices used in antimicrobial stewardship programs in high-income countries. We recommend interrupted time series studies be used as an alternative design to pre- and post-intervention studies, information about the clinical guidelines be made more transparent, and prescriber confidence be investigated.

## 1. Introduction

Curbing rising levels of antimicrobial resistance (AMR) and maintaining the effectiveness of antibiotics are major global public health concerns. The inappropriate use of antibiotics is a key driver of AMR. Research assessing global trends in antibiotic consumption found total consumption (defined daily doses (DDDs) per 1000 inhabitants per day) increased 65% between 2000 and 2015 and the rate of antibiotic consumption by 39% (DDDs) [1]. In LMICs the increased consumption rate was substantial: (77% (DDDs) and is explained by rising income levels due to rapid economic growth which is providing greater access to antibiotics. A greater proportion of this increase in antibiotic consumption was accounted for by low- and middle-income countries (LLMICs) than by upper middle-income countries (UMICs). Total antibiotic consumption increased by 117% (8.1 to 17.4 billion DDDs) in LLMICs compared with 110% (3.3 to 6.9 billion DDDs) in UMICs [1]. However, high populations in some LLMICs (i.e., India) result in greater total antibiotic consumption even though rates of consumption are lower.

In 2015, the World Health Assembly endorsed the Global Action Plan (GAP) to contain AMR [2]. The GAP called for all countries to develop a National Action Plan (NAP) in line with the GAP strategic objectives that accounts for each country’s unique circumstances. Guided by the World Health Organization (WHO), LLMICs have been developing NAPs, but progress on implementation has been slow. LLMICs are hindered by insufficient resources, weaker health systems, limited access to reliable antibiotic susceptibility testing and guidance on appropriate antibiotic use and, lack awareness of AMR [3,4]. Thus, whilst the need to develop NAPs to preserve the effectiveness of antibiotics is paramount, it is challenging in LLMICs.

One of the GAP strategic objectives includes optimising the use of antimicrobial agents through antimicrobial stewardship [2]. This objective emphasises the need to establish antimicrobial stewardship programmes (ASPs). An ASP is defined as an organisation-wide strategy to promote appropriate use of antimicrobials through the implementation of evidence-based interventions [5]. Guidelines and standards underpin ASPs and LLMICs are developing and implementing clinical guidelines (CGs), albeit slowly [4,6]. Clinical guidelines are defined as “statements that include recommendations intended to optimize patient care that are informed by a systematic review of evidence and an assessment of the benefits and harms of alternative care options” [7]. It is well documented that using evidence-based CGs targeting prescribing practices can reduce antimicrobial consumption and improve patient outcomes [8,9,10]. However, the degrees to which CGs are adopted and the recommendations adhered to continues to challenge developers, policy makers and CG users [9,10,11].

A systematic meta-review examining factors influencing CG implementation reported degree of complexity as the most frequently cited characteristic affecting compliance [12]. Clinical guidelines which are easy to comprehend and incorporate into daily practice have a greater chance of being used [12]. Other influential CG characteristics include strength of evidence, relevance to practice, format and online availability [13].

Clinical guideline implementation strategies which have been used to improve prescribing practices for infectious diseases in HICs and have resulted in some measure of success have included: academic detailing (face-to-face educational outreach for prescribers by health professionals); audit and feedback; small group discussions; feedback with reminders; and communication skills training [10,13,14,15]. These strategies have been used either alone or combined in a multifaceted intervention which is assumed to be more effective [7]. However, the findings of research into whether multifaceted interventions are more effective for creating behaviour change than single interventions are mixed [12,14,16,17].

There are numerous published systematic reviews about interventions to improve CG compliance and antibiotic prescribing that have included UMICs [15,18,19]. However, this is not the case for LLMICs. One systematic review, devoted to LLMICs and published prior in 1999 was identified [20]. There is scarce knowledge about CG uptake and adherence in LLMICs since 2000. Therefore, this review aims to highlight research conducted in LLMICs into strategies for implementing and promoting adherence to CGs from 2000 to 2020

## 2. Materials and Methods

Our review protocol was registered with PROSPERO (CRD42020153918) and our review is reported according to the preferred reporting items for systematic reviews and meta-analysis (PRISMA)checklist (supplementary file Table S4) [21].

### 2.1. Terminology

In this review CGs include therapeutic guidelines, standard treatment guidelines and clinical practice guidelines. Treatment refers to antibiotic treatment (i.e., the use of antibacterial agents to treat infections in humans). We have used the term antibiotic rather than the broader term antimicrobial because the burden of AMR is with bacteria in LLMICs and, the review only focussed on bacteria. 

### 2.2. Search Methods for Identification of Studies

We conducted literature searches using PubMed, Cochrane Library, SCOPUS and the information systems of the Australian National University and the WHO website. Google Scholar was searched for additional studies. We developed search strategies for the PubMed database which were translated using appropriate syntax, subject headings and vocabulary for the other databases. The reference lists of the results retrieved were searched manually for additional items. Search strategies for PubMed can be found in supplementary file Table S1.

### 2.3. Study Screening and Inclusion/Exclusion Criteria

The titles and abstracts of the search results were subjected to an initial screen for potential eligibility by one reviewer (N.F.). Two reviewers, (N.F., C.H.) read the full text of the selected studies independently and assessed the studies for inclusion by applying the inclusion and exclusion criteria. A third reviewer (N.T.) made the final decision in cases where agreement could not be reached. 

#### 2.3.1. Inclusion

- Randomised controlled trials (RCTs), cluster RCTs, controlled clinical trials, quasi experimental studies, controlled pre- and post-intervention studies (CPPI), pre- and post-intervention studies (PPI) and interrupted time series (ITS) studies.
- The LLMICs include those listed by the Organisation of Economic Cooperation and Development’s (OECD) Development Assistance Committee for 2018 to 2020 [22].
- Health workers in LLMICs who prescribe antibiotic therapy.
- Patients from all age groups in LLMICs who receive antibiotic therapy.
- Any strategy which was aimed at promoting CG uptake or compliance for the purpose of improving rational antibiotic prescription.
- Studies published in English language between 2000 and July 2020.
- The primary outcomes included health worker performance based on appropriateness of prescribing including:
  ■ Correct agent, correct dose, correct duration, correct route of administration or time of administration.
  ■ Proportion of antibiotics prescribed in accordance with CG.
  ■ Consumption of antibiotics expressed as defined daily doses per 100 or 1000 patient days.
  ■ Patient encounters with an antibiotic.
  ■ Patient outcomes—mortality and hospital re-admission rates.
  ■ Adverse effects impacting patient outcomes.

#### 2.3.2. Exclusion

- Commentaries, conference proceedings and literature reviews
- Languages other than English.

### 2.4. Study Quality Assessment

The included studies were appraised for risk of bias using one of two risk of bias tools. The Downs and Black Risk of Bias tool was used for all studies except the ITS studies [23]. The ITS studies were appraised using criteria specifically developed by Ramsay et al. to strengthen reviews which include ITS designs [24]. Two reviewers independently applied the risk of bias tools (N.F., C.H.). When discrepancies occurred, the independent assessment of a third reviewer (K.R.) provided consensus. The criteria comprising the risk of bias tools were used to calculate the scores for high, medium, and low risk for each study. 

### 2.5. Data Extraction Method

An Excel spreadsheet was used by the first author for extraction and storage of the data. The following information was recorded about each study: author, date of publication, study title and design, country where the study took place, description of context, aim of study, number and age group of participants, period of study, infectious disease focus, CG source, intervention description, data summary, outcome measure and effect size. Tables were prepared to display the information which was reported according to study design. 

### 2.6. Data Synthesis and Presentation

The selected studies varied widely in study design and quality, range of intervention components, settings, infectious disease focus and measures of effect. A bubble plot (supplementary Figure S1) was used to display study diversity using the number of intervention components (strategies) implemented per study on the y axis and studies by design type identified by bubble colour on the x axis with study quality indicated by bubble size [25]. Given the diversity of the studies and the complex nature of the interventions, our results are presented using a narrative synthesis approach, supported by an effect direction plot [26,27].

The CGs were classified into groups based on an approach devised by Dizon, JM et. al. [28]. to customise a CG to suit local conditions: developed from scratch by local health workers and obtained from the WHO, other international organizations or medical associations. The latter group was further divided into CGs which were adopted and CGs which were adapted. An adopted CG was implemented in its entirety with some contextualisation to suit local issues (i.e., staffing, patient’s access to care, training). If the CG was adapted, the recommendations were modified to suit the local environment (e.g., need to substitute one antibiotic for another due to cost and availability). Whilst every effort was made to assign the CGs to one of the groups, there was limited information about the source of the CG in some studies, hence an additional category, the CG was pre-existing with no additional information included.

The Cochrane Effective Practice and Organisation of Care (EPOC) taxonomy for implementation strategies was deemed an appropriate tool to use to develop broad categories for grouping and displaying the intervention strategies in a table [18,29]. The EPOC taxonomy has been applied to meta-reviews of health system interventions which are relevant to LLMICs [18]. Supplementary file Box S1. provides a detailed explanation of these categories and the strategies included in each category.

The methods used to measure the intervention outcomes in the studies were grouped into five broad outcome domains: (i) encounters with an antibiotic; (ii) antibiotics prescribed appropriately: dose, timing, and duration; (iii) defined daily doses per 100 bed-days; (iv) rate of clinical failure; and (v) CG indicator scores. Clinical guideline indicators were used to measure appropriate antibiotic use and practical competencies in clinical examination, diagnosis and treatment. The domains were used to develop an effect direction plot to synthesise and report the direction of study effects [27]. The effect direction plot provides a method of synthesising the data when meta-analysis is not possible [27]. The evidence for improvement, deterioration or no change/mixed effects indicated by each study’s primary intervention outcome is represented by the use of up, down or bi-directional arrows. The nonparametric sign test was used to support the synthesis of effect direction across outcome domains not limited by a small number of studies (≤5). The sign test includes studies with positive or negative effect direction for an outcome domain. Studies with unclear/mixed or no effect were excluded. The power of the sign test is limited when the number of studies included in a domain is small because the number of studies may be further reduced when those with unclear/mixed or no effect are excluded [29].

## 3. Results

### 3.1. Description of the Studies

#### 3.1.1. Search Results and Study Quality

**Figure 1:**
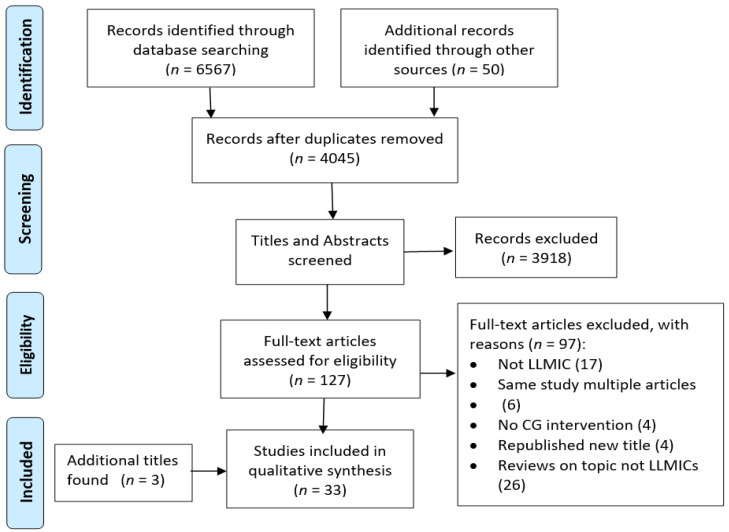
The preferred reporting items for systematic reviews and meta-analysis (PRISMA) flow chart detailing the selection of studies investigating strategies used for implementing and promoting adherence to antibiotic guidelines in low- and lower middle-income countries (LLMICs).

The database search yielded 6567 articles. After removing duplicates, we screened 4045 titles and abstracts, selected 127 full text articles to review and included 33 studies in our systematic review (Figure 1). The study designs included five RCTs, five cluster RCTs, two quasi-experimental studies, five ITS studies, three CPPI studies and 13 PPI studies. Five studies had been included in an earlier systematic review [19]. Overall, the quality of the studies was generally low with only 15% (*n* = 5) scoring low risk of bias. Three RCTs, one cluster RCT and one PPI study were assessed as low risk. Medium risk of bias was attributed to 42% (*n* = 14) of studies and 42% (*n* = 14) scored high risk of bias. The main risks were related to internal validity: the lack of a control group (*n* = 13); the selection of participants into the study was non-randomised; and the lack of clarity around how the interventions were assessed (*n* = 5). The authors who used a PPI study design did not report on attempts to address threats inherent in the design (e.g., unrelated historical events, dropouts, Hawthorne effect). Few studies reported on measures taken to ensure persons measuring the research outcomes were blinded (*n* = 5). Three studies were limited by small sample sizes and in one study no sample size was provided. The risk of bias results for all studies can be found in supplementary file Table S2a,b.

#### 3.1.2. Settings and Study Participants

The studies were conducted in 16 countries: Afghanistan (1) [30], Bangladesh (4) [31,32,33,34], Egypt (1) [35], Ethiopia (1) [36], India (7) [37,38,39,40,41,42,43], Indonesia (2) [44,45], Kenya (3) [46,47,48], Lao PDR (2) [49,50], Nepal (2) [51,52], Pakistan (1) [53], Serra Leone (1) [54], Sri Lanka (1) [55], Sudan (1) [56], Tanzania (2) [57,58], Vietnam (3) [59,60,61] and Zimbabwe (1) [62]. More than two thirds (68%; *n* = 23) of the studies were conducted in hospital settings, 26% (*n* = 9) in community health clinics or rural health centres and 6% (*n* = 2) were drawn from both hospital and community. Over one third of the studies (38%; *n* = 13) focused on paediatric patients. One study was devoted entirely to female patients aged ≥ 14 years whilst other studies were mixed gender. Overall, most studies 73% (*n* = 24) included more than 500 participants in the experimental or intervention group. Two studies included between 500 and 300 participants, six studies included less than 300 and this detail was not provided in one PPI study. Further details can be found in Table 1 which provides a description of the studies. 

All studies reported on antibiotic prescribing in association with one or a combination of infectious diseases. Diarrhoeal disease was investigated in seven studies and, acute respiratory tract infections (ARTIs), healthcare-associated infections (HAIs) and hospital ASPs were examined in six studies each. One study focussed on community-acquired (CA) urinary tract infections (CA-UTIs) and the remaining studies investigated strategies to optimise antibiotic prescribing without specifying a particular type of infection.

#### 3.1.3. Clinical Guidelines

Seventy-nine percent (*n* = 26) of studies provided information about the origin of the CG. The CGs were developed from scratch in 28% (*n* = 9) of studies. Twenty-one percent (*n* = 7) and 30% (*n* = 10) of studies, respectively, reported adopting and contextualising the CG or adapting a guideline that had been developed by WHO, other international organizations or medical associations. The remaining 21% (*n* = 7) referred to CGs which were already in place at the time of the study and information about their origin was not reported. In eight studies prescribers were either key participants in CG development or were invited to contribute feedback during the process. 

### 3.2. Interventions

#### 3.2.1. Strategies Used

There were 18 different strategies implemented to increase compliance with CG recommendations across the 33 studies. These 18 strategies were classified into five broad categories: organisational, capacity building, monitoring and review, clinical decision support systems (CDSS) and persuasive strategies. Table 2 details the categories and associated strategies used either singularly or as components of multifaceted interventions in each study to promote and improve CG adherence. 

The interventions in 85% of studies (*n* = 29) were multifaceted, combining strategies from either the same category or across categories. Three or more categories were combined in 27% (*n* = 9) of studies, 58% (*n* = 19) combined two categories and the remaining five studies investigated strategies from one category each. Most (70%) studies employed strategies from the categories of capacity building (*n* = 23) or monitoring and review (*n* = 23). Organisational and CDSS strategies were implemented in 38% (*n* = 13) and 42% (*n* = 14) of studies, respectively. Persuasive strategies were used in just two studies. Only one study which implemented a multifaceted intervention compared the impact of the individual strategies. 

#### 3.2.2. Organisational Strategies 

Organisational strategies including management endorsement, stakeholder consensus, engaging a champion, institutional incentives and hospital ASPs were all components of multifaceted interventions. Clinical guidelines which had management endorsement (*n* = 3) were made compulsory. 

Studies which obtained stakeholder consensus for the CG (*n* = 6), achieved this by involving prescribers in CG development. Researchers in Lao PDR involved the Lao Paediatric Network in translating the guideline, the *WHO Pocketbook of Hospital Care for Children,* into the local language and then engaged local opinion leaders amongst the Network to help drive the intervention [49]. Likewise, in an Indian study using the Plan, Do, Study, Act (PDSA) model to improve prescribing for URTIs, staff feedback was obtained during initial awareness-raising discussions about the problem and channelled into follow-up training. Subsequently, the quality improvement team collaborated with staff to design best practice recommendations for the new CG [39]. Two studies reported using champions to advocate for the CG. In a study in Nepal seven physician champions, one for each department, were specially trained to conduct the intervention in their departments. Their tasks included audit and feedback; modify/de-escalate/stop treatment according to the CG; attend all staff training; and keep a log of all associated activities [51]. In an Egyptian study, senior surgeons were nominated as champions to audit prescriptions in patient charts and give one-to-one feedback about the prescription plan with the prescriber when needed [35].

Hospital-based ASPs were reported in six studies. The ASP committees drove the interventions which included one or more activities from across the categories. The following three studies provide examples. In Egypt, the infection control teams in seven hospitals delivered training workshops and conducted audit and feedback to improve adherence to surgical prophylaxis [35]. In Indonesia, HAIs and prescribing were assessed after a new CG and hand hygiene campaign, including educational seminars, reminders and weekly audit and feedback had been delivered by infection control staff [45]. Pharmacists were also involved in leading ASP interventions. In Ethiopia, a CG based on the latest antibiogram data was loaded onto a mobile stewardship application. Pharmacists supported by laboratory staff conducted training in AMR, antibiotic prescribing, laboratory services and report interpretation and then carried out weekly AMS audit and feedback rounds [36]. 

#### 3.2.3. Capacity-Building Strategies

Capacity-building strategies involved developing prescriber skills and competencies. Workshops and seminars were used in 91% (*n* = 21) of studies, focus group discussions in 35% (*n* = 8) and one third of the studies (*n* = 7) combined both strategies. Follow-up training was used in three studies and academic detailing (onsite training and follow-up by a clinical expert) in just two. In total, 33% (*n* = 12) of studies in the capacity-building category found positive effect change in the use of antibiotics and CG adherence.

The following studies illustrated how these activities were used. In Laos, an intervention conducted across seven hospitals included regular educational presentations, group discussions focusing on diagnosis and treatment of key infectious diseases and follow-up audit and feedback [50]. Two studies combined training seminars with face-to-face educational outreach by a pharmacist and one added supervision of the CG implementation to the mix [48]. A multi-disciplinary panel of experts in Kenya adapted a CG for the treatment of CA-UTI and developed indicators for quality of care for screening, diagnosis and treatment to measure compliance over a nine-month period. The multifaceted intervention comprised interactive educational workshops based on the CG, peer-to-peer review and feedback of patient charts, according to CG compliance and focus group discussions on research into AMR patterns in uropathogens. The primary outcome—appropriate antibiotic prescription—improved from 19% at baseline to 68% by the end of the study period [47]. 

A study from Sudan combined both capacity-building and monitoring and review strategies and, unlike other studies in this review, measured the impact of the components [56]. When audit and feedback, a monitoring and review strategy was used alone antibiotic consumption did not change. However, audit and feedback coupled with either academic detailing or educational seminars showed positive effect change at both one- and three-month post-intervention. [56] Further details of the outcome results can be found in supplementary file Table S3a,b.

#### 3.2.4. Monitoring and Review Strategy

Monitoring and review strategies were frequently components of study interventions (n = 23). Audit and feedback and practice supervision were components of 13 and seven studies, respectively. Antimicrobial restriction was used in four studies and reminders in two. Fourteen of the studies also incorporated a capacity building strategy and 64% (*n* = 9) of these showed positive effect change in CG uptake. Audit and feedback was coupled with focus group discussions (*n* = 6) or workshops and seminars (n = 6) or both (n = 5). All, but one study using practice supervision (*n* = 7) was combined with workshops and seminars (n = 6). 

The following examples demonstrated the use of monitoring and review activities. A RCT in Zimbabwe investigated supervision, together with audit and feedback across different types of infections [62]. Pharmacy staff attended 14 days of training in the theory and practice of supervision before auditing prescriptions and holding on the spot discussions with health workers to improve knowledge and performance. The results were mixed: CG adherence improved in treatment for non-bloody diarrhoea and ARTI, but not genital infections. An antibiotic restriction policy was used in four studies [37,41,52,53]. All were implemented in a similar way. The policies involved completing an antibiotic justification form before commencing use of a restricted antibiotic and, if after reviewing the culture report, it was decided to continue treatment, approval had to be sought from a senior clinician or ASP committee member. Three of the studies found a reduction in the use of antibiotics. Additional information about the results can be found in supplementary file Table S3a,b.

#### 3.2.5. Clinical Decision Support Systems

Forty-one percent (*n* = 14) of studies used CDSS strategies. The WHO’s clinical algorithm, ALMANACH (Algorithms for the Management of Acute Childhood illnesses) was used in three studies [30,57,58]. Two of the studies, one in Afghanistan and the other in Tanzania adapted ALMANACH for use on mobile technology [30,58]. A third study, also from Tanzania, compared ALMANACH with ALMANACH and e-POCT, a smartphone-based algorithm that incorporated point of care tests (oximetry, haemoglobin, C-reactive protein, and procalcitonin) for the management of febrile illness [57]. Rapid diagnostic testing tools were also used to promote the rational use of antibiotics in influenza-like illness, acute respiratory tract infections and diarrheal disease [33,55,60]. All involved smartphone technology, but the level of training clinicians received varied. In a study from Sri Lanka clinicians received no training but were referred to the CG disseminated by the Ministry of Health [54]. Clinicians in a Vietnamese study attended initial workshop presentations in a central location and follow-up onsite training with leaflets and posters and were given a telephone contact number for further queries [60]. All of the studies found positive improvement in adherence to CG recommendations.

Quick reference material was reported as an intervention component in 24% of studies (*n* = 8). This included wall charts, leaflets, posters, drug lists and booklets available in print, on stand-alone computers, hand-held devices or via hospital intranets. Only one study, an Indian ITS study used multiple disseminations of the CG as the intervention strategy. The CG was distributed four times following initial stakeholder participation in development, revisions to content and changes in format. Adherence improved after the CG was made available online during the final stage of development [38].

#### 3.2.6. Persuasive Strategies

Only two studies used persuasive strategies: one through peer pressure, and the other by way of formal contractual obligations [43,59]. In an Indian study, the prescribing decisions of each clinician were considered to be a group decision of the unit. Each unit (*n* = 35) received a monthly prescribing score based on the amount of antibiotics consumed relative to that consumed by all units. Scores were then shared across all units and discussion followed. At three-months post-intervention 43% of units (*n* = 15) had reduced their antibiotic consumption [43]. In the Vietnamese study, health officials were required to sign contracts and pledge their commitment to carry out supervision. Staff received training and reminders and were required to support the CG. Funds and equipment were donated to community health hubs on the condition monthly supervision was deemed adequate, and prescribing had improved. The primary outcome, adequate antibiotic dose improved from 30% to 90% post-intervention [59].

### 3.3. Outcomes

Table 3 displays the effect direction plot that summarises the direction of effects of the intervention outcomes according to outcome domain for all studies according to risk of bias score.

Overall, 67% (*n* = 22) of the studies provided evidence to indicate improvement in CG adherence: the outcome effects indicating a positive direction. The study designs in this group included four RCTs, one cluster RCT, two QE studies, two ITS studies, three CPPI and 10 PPI studies. The outcomes of 33% (*n* = 11) of the studies indicated either mixed (*n* = 5), unclear (*n* = 3) or no change in effects (*n* = 3), thus, suggesting no overall change in CG adherence. This group included one RCT, four cluster RCTs, three ITS studies and three PPI studies.

The effect direction plot displayed in Table 3 shows all studies reported a positive direction of effect for measures of reduction in encounters with an antibiotic with four studies reporting no change or unclear effect. The p-value for the sign test for this domain is 0.0005 at the 0.05 level. The nine studies which scored either low or medium risk of bias for this domain show a positive direction of effect with one study indicating no change or unclear effect and the *p*-value of 0.0027 at the 0.05 level is slightly lower than that when all studies are included. For antibiotics prescribed appropriately according to dose, timing, and duration, the 10 included studies reported a positive effect direction with one finding a negative effect and four studies finding mixed or unclear effects (*p*-value for the sign test is *p* 0.0066). When the seven studies which scored high risk of bias were excluded, five studies showed a positive direction of effect, one a negative direction and one study reported no change or unclear effect (*p*-value 0.1024 at the 0.05 level). Studies with no change/unclear effect could not be included in the sign tests. The three remaining domains: reduction in defined daily doses per 100 bed-days; reduction in clinical failure; and CG knowledge scores all show studies with positive directions of effect, however, the total number of studies in these categories is too small (<5) to apply the sign test to. For further information about the study outcome measures and effect sizes see supplementary file Table S3a,b which provides a summary of outcome effects as reported by the authors.

## 4. Discussion

Our review identified 33 studies from 16 LLMICs that were published between 2000 and 2020 and examined strategies for implementing and promoting antibiotic guidelines. This collection of studies represents just 21% of LLMICs. The studies varied widely in terms of design, settings, target groups, strategy types, intervention components and timeframes, implementation methods, outcome measures and effects. The quality of the studies was generally poor with 40% using an uncontrolled pre- and post-intervention design, many of which, scored a high risk of bias. 

It was not possible to tease out which strategies had the greatest impact on improving CG compliance, because of the complex nature of the interventions. Interventions in the majority of studies were multifaceted and only one study with a multifaceted intervention assessed the individual components. Therefore, for most studies, it was not possible to assess the contribution any one strategy made to the outcome or to establish how vital a single strategy was to the success of the entire intervention. The Institute of Medicine (USA) and others recommend employing multifaceted interventions over single strategies to promote adherence to CG [7,63,64]. However, whilst there is evidence for the effectiveness of particular strategies, such as audit and feedback, antimicrobial restriction or reminders, when it comes to which specific components are associated with increased effectiveness in a multifaceted intervention, the evidence is not available. 

Clinical decision support systems which used digital technology to deliver the intervention, stood apart from other strategies. Rapid diagnostic testing tools and digital algorithms employed smartphone technology, were implemented with little or no support from other strategies, and all found a measure of improvement in antibiotic use. In LLMICs where access to laboratories is limited, broad spectrum antibiotics are routinely used empirically for patients presenting with acute fever, for example. Ascertaining whether fever symptoms are the result of a bacterial infection based on clinical presentation alone is challenging. However, using antibiotics unnecessarily drives AMR. Thus, POCTs have the potential to reduce antibiotic consumption by supporting prescribing decision-making in LLMICs. A qualitative study in South Africa reported most clinicians regarded POCTs as having potential for common infections: aiding diagnosis, indicating when an antibiotic is not needed, enabling earlier treatment and managing patient expectations. However, resource issues were identified as a barrier [65]. In 2019, the WHO reviewed the first Essential In Vitro Diagnostics (IVDs) List (EDL) to provide guidance to Member States developing interventions for EDLs and for selecting and using IVDs [66]. Access to digital technologies is growing in LMICs: the median rate of smart phone ownership was 37% in 2015, having risen from 21% in 2013 [67]. However, whilst digital technologies have the potential to transform the delivery of health care in resource-poor settings, major challenges (e.g., funding, ownership, privacy) need to be overcome. 

Antimicrobial stewardship programmes were implemented in just six studies. Even though progress is slow, the evidence suggests LLMICs are moving towards implementing ASPs by engaging with ASP strategies in their efforts to reduce antibiotic consumption and improve CG compliance [19]. A wide range of intervention strategies were implemented, and all are used in ASPs in HICs. Most of the interventions were driven by senior clinicians, infection control experts and pharmacists. This is similar to the approach taken in HICs where a multi-disciplinary team of experts is an integral component of an ASP [68]. It is noteworthy that the studies reporting on ASPs called for further research into ASPs: initiating, implementing and maintaining ASPs, benefits of ASPs and involving pharmacists in ASP initiatives. 

At least one third of CGs were developed from scratch. Developing CGs based on evidence is time-consuming, costly and requires research expertise and commitment to keep the recommendations up to date [69]. Achieving all of these elements may be overly ambitious for many LLMICs. Improving access to freely available trustworthy evidence based CGs from international guideline repositories (NHMRC, NICE), medical associations and WHO will benefit LLMICs. Regardless of the source, CGs must suit the local situation, be trusted by the end-users and easily accessible. Only eight studies reported engaging stakeholders in CG development. There is an association between barriers to uptake (e.g., complexity, end user trust in the CG) and stakeholder participation in CG development [12,41]. Stakeholders differ in education and experience. Therefore, involving stakeholders in CG development, evaluation and implementation, allows for trust and a sense of ownership to be built, and differences to be accommodated [16,41]. 

Mobile applications (apps) are commonly used to provide access to evidence-based CG in HICs, though little is known about this in LLMICs. A recent study in four African countries investigated prescriber perceptions and assessment of CGs used on a smartphone app [70]. Prescribers (*n* = 38) reported that the app increased their awareness of antimicrobial stewardship, was the “best way” to access CGs, caused them to re-appraise their prescribing as well as document the patient drug chart. Further research into the use and effectiveness of mobile technology for CGs in resource-poor settings is needed [70].

There are several limitations to this work. The outcomes measured were limited; only three studies examined clinical failure and no studies reported on prescriber confidence even though several investigated capacity building strategies. Slightly more than half of the studies overall found a positive direction of effect in guideline adherence, suggesting the research on this topic may be limited by publication bias. It is not unexpected that the studies finding positive effect change also included three-quarters of the studies which used the bias-prone PPI design [24]. The more robust ITS design, which collects data at multiple time-points before and after the intervention is implemented is recommended as an alternative to the PPI design [24]. The inclusion criteria allowed for a wide range of study designs, strategies, settings, methodologies, outcomes and CG foci. Heterogeneity is often cited as a characteristic of systematic reviews and hinders meta-analysis being conducted, comparisons being made, and the ability to generalise from the results [19,71]. 

## 5. Conclusions

In this review most interventions showed a positive direction of effect. It was not possible to recommend any one strategy or combination of strategies in the selection of intervention components to improve uptake of CGs in LLMICs, because of the complex nature of the interventions and the limitations of the studies, even though some strategies were particularly notable. Audit and feedback coupled with either educational workshops and/or focus group discussions were the most frequently used intervention components. Clinical decision support systems which made use of mobile technologies proved they could be implemented with little or no support from other strategies. The implementation of ASPs remains slow in LLMICs; however, LLMICs are moving in the right direction by engaging with antibiotic stewardship strategies. Our review suggests other LLMICs need to conduct similar studies. We recommend ITS studies be used as an alternative design to PPI studies, information about the CG be made more transparent, and prescriber confidence be investigated to aid future research

## Figures and Tables

**Table 1:** Description of studies included in systematic review investigating strategies for implementing and promoting adherence to antibiotic guidelines in LLMICs.

Author (Year)	Titles	Country	Description of Context	Participants	Study Length; Outcome Measure
Randomised Control Trials					
Keitel, K. et al. (2017) [57]	A novel electronic algorithm using host biomarker point-of-care tests for the management of febrile illnesses in Tanzanian children (e-POCT): A randomized, controlled non-inferiority trial.	Tanzania	Outpatient departments at 3 district hospitals and 6 health centre clinics in Dar es Salaam; in parallel, routine care documented in 2 additional health centres.	3716 paediatric patients: age 24 to 59 months with acute febrile illness (≤7 days fever ≥37.5 °C temperature): ALMANACH plus e-POCT: 1586 (intervention) and ALMANACH alone: 1583 (control); 547 in parallel control.	• 13 months long; • No. clinical failures day 7; • No. prescriptions day 0; • No. primary referrals.
Do, N.T.T. et al. (2016) [60]	Point-of-care C-reactive protein testing to reduce inappropriate use of antibiotics for non-severe acute respiratory infections in Vietnamese primary health care: a randomised controlled trial.	Vietnam	9 urban polyclinics and the outpatient clinic of a rural district general hospital within a 60 km radius of Hanoi.	2037 patients presenting with non-severe ARTIs: 1017 intervention and 1019 control group.	• 15 months long; • No. prescriptions within 14 days; • Antibiotics in urine days 3/4/5.
Shao, A.F. et al. (2015) [58]	New Algorithm for Managing Childhood Illness Using Mobile Technology (ALMANACH): A controlled non-inferiority study on clinical outcome and antibiotic use in Tanzania.	Tanzania	Two pairs of primary healthcare facilities, 1 pair in urban Dar es Salaam and 1 pair in rural Morogoro with similar catchment populations and services.	Paediatric patients 2 to 59 months; Total 842 in intervention (ALMANACH) and 623 in control group. Illness not reported.	• 7 months long; • No. prescriptions day 0; • Cured day 7.
Trap, B. et al. (2001) [62]	The impact of supervision on stock management and adherence to treatment guidelines: a randomized controlled trial.	Zimbabwe	62 rural health centres across 7 of the 8 Provinces randomized into 3 groups: 21 to stock management; 23 to standard treatment guideline and 18 to a control group.	10 pharmacy officers and 1–2 nursing staff per rural health centre. Illness: non-bloody diarrhoea; ARTIs, genital ulcer.	• 12 months long; • No. correct drug, dose, duration.
Wahlstrom, R. et al. (2003) [50]	Effectiveness of feedback for improving case management of malaria, diarrhoea and pneumonia—a randomized controlled trial at provincial hospitals in Lao PDR.	Lao PDR	8 Provincial hospitals in the Vientiane Municipality—24 departments matched into 4 pairs of 3 departments: internal medicine, paediatrics and outpatients and randomised to intervention and control groups	Prescribers: doctors and medical assistants: 53 in intervention and 69 in control. Illness: diarrhoeal disease, malaria, pneumonia.	• 12 months long; • Changes in key CG indicator scores.
**Cluster Randomised Control Trials**					
Awad, A.I. et al. (2006) [56]	Changing antibiotics prescribing practices in health centres of Khartoum State, Sudan.	Sudan	20 health centres in Khartoum State: 1 control and 3 intervention groups.	1800 patient encounters; 1 general practitioner and 1 or 2 medical officers per health centre. Illness not reported.	• 6 months long; • % Encounters with antibiotics.
Chowdhury, A.K. et al. (2007) [32]	Effect of Standard Treatment Guidelines with or without Prescription Audit on Prescribing for Acute Respiratory Tract Infection and Diarrhoea in some Thana Health Complexes of Bangladesh.	Bangladesh	24 randomly selected health complexes from 120 Thana health complexes, primary health centres in Dakar district: 2 groups of 8 in intervention and 1 group of 8 in control.	6000 prescriptions collected baseline and total number post-intervention not reported. Illness: ARTIs and diarrhoeal disease.	• 7 months long; • % Encounters with antibiotics.
Hoa, N.Q. et al. (2017) [61]	Antibiotic prescribing and dispensing of acute respiratory infections in children: Effectiveness of a multi-faceted intervention for health-care providers in Vietnam.	Vietnam	A rural 150-bed district hospital, 3 regional polyclinics and 32 commune health clinics in Bavi district north of Hanoi; randomised to 2 arms: STIs and ARTIs.	Total of 299 health care practitioners: 139 pre- and 160 post-intervention. Illness: mild ARTIs. Total prescriptions: 1279 intervention; 742 control.	• 7 months long; • Mean KAP scores; • % Appropriate prescription.
Opondo, C. et al. (2011) [48]	Effect of a multi-faceted quality improvement intervention on inappropriate antibiotic use in children with non-bloody diarrhoea admitted to district hospitals in Kenya.	Kenya	8 district hospitals randomised into 2 groups of 4 across 4 Provinces.	130 health workers in 4 intervention group hospitals and 135 health workers in: 4 control; 1160 admission records of children < 5years old with non-bloody diarrhoea not requiring antibiotics.	• 36 months long; • No. inappropriate prescriptions received.
Shrestha, N. et al. (2006) [52]	World Health Organization’s (WHO) ractical approach to lung health in Nepal: better prescribing and reduction of cost.	Nepal	40 primary health facilities randomized into 19 control groups and 21 intervention groups.	84 patients pre- and 67 post-intervention in the control group and 155 pre- and 101 post-intervention in the intervention group. Illness: lung disease.	• 12 months long; • % Encounters with antibiotics; • Adherence to WHO’s PAL.
**Interrupted Time Series Studies**					
Aiken, A.M. et al. (2013) [46]	Changing use of surgical antibiotic prophylaxis in Thika Hospital, Kenya: a quality improvement intervention with an interrupted time series design.	Kenya	Public hospital with 300 beds 50 km northeast of Nairobi performing 300 surgical procedures monthly.	3343 patients undergoing surgical procedures involving overnight stay; 6 surgeons and 16 to 20 junior doctors and clinical officers. Illness: SSIs	• 16 months long; • 66 data points • % Operations with correct prophylaxis.
Chalker, J. (2001) [59]	Improving antibiotic prescribing in Hai Phong Province, Viet Nam: the antibiotic-dose indicator.	Vietnam	217 commune health stations in 12 Provincial Districts.	6270 records examined; total number health workers not reported. Illness not reported.	• 18 months long; • 6 datapoints • % Encounters with antibiotics; • % Receiving adequate dose.
Chandy, S.J. et al. (2014) [38]	The Impact of Policy Guidelines on hospital antibiotic use over a decade: A segmented time series analysis.	India	2140-bed teaching hospital in south India serving 6000 outpatients per day with Drugs and Therapeutics Committee and Formulary Sub-Committee.	All inpatients in the hospital over a 10-year period prescribed an antibiotic. Illness: not reported.	• 10 years long; • 110 datapoints • DDD per 100 bed-days.
Hadi, U. et al. (2008) [44]	Optimizing antibiotic usage in adults admitted with fever by a multifaceted intervention in an Indonesian governmental hospital.	Indonesia	Internal medicine department of a 1432-bed teaching hospital with 60,000 annual admissions.	501 patients’ consultations within first 24 hours of admission; 155 clinicians. Illness: fever.	• 1 year long; • 28 datapoints • DDD per 100 bed-days.
Wattal, C. et al. (2017) [43]	Antimicrobial prescribing patterns of surgical speciality in a tertiary care hospital in India: Role of persuasive intervention for changing antibiotic prescription behaviour.	India	45 clinical units across a 675-bed tertiary hospital in New Delhi.	90 clinicians, all prescribers across all specialities. Illness: SSIs.	• 9 months long; • DDD per 100 bed-days.
**Quasi-Experimental Studies**					
Gebretekle, G.B. et al. (2020) [36]	Half of prescribed antibiotics are not needed: a pharmacist-led antimicrobial stewardship intervention and clinical outcomes in a referral hospital in Ethiopia.	Ethiopia	Referral and teaching hospital in Addis Ababa, with 800 beds (191 paediatric beds)	1264 prescriptions (707 patients) intervention and 1138 (402 patients) post-intervention. Illness: sepsis, febrile neutropenia, CA & HA pneumonia.	• 15 months long; • Mean no. days patient receives antibiotic; • Days of therapy per 1000 bed-days.
Sarma, H. et al. (2019) [34]	Evaluating the use of job aids and user instructions to improve adherence for the treatment of childhood pneumonia using amoxicillin dispersible tablets in a low-income setting: a mixed method study.	Bangladesh	Community health centres and Union health and family welfare centres in Ghatail and Kalihati sub-districts: 55 and 17; 54 and 18, respectively	94 health workers: 56 in intervention group; 38 in control group. Illness: pneumonia.	• 4 months long; • % Receiving appropriate antibiotic prescription & treatment.
**Controlled Pre- and Post-intervention Studies**					
Akter, S.F. et al. (2009) [31]	Impact of a training intervention on use of antimicrobials in teaching hospitals.	Bangladesh	3 medical college hospitals providing tertiary care and referral to secondary and primary level hospitals: 1 intervention and 2 control.	3466 paediatric patients receiving antibiotics: 2171 pre- and 1295 post-intervention. Illness: pneumonia, diarrhoeal disease.	• 12 months long; • % Receiving appropriate antibiotic prescription & treatment.
Bernasconi, A. et al. (2018) [30]	Can the use of digital algorithms improve quality care? An example from Afghanistan.	Afghanistan	3 Afghan Red Cross Society health centres in Kabul Province.	767 paediatric patient: age 2–5 years.: 404 pre- and 362 post-intervention. Illness: acute childhood illness	• 16 months long; • % Receiving appropriate antibiotic prescription & treatment.
Haque, F. et al. (2017) [33]	Evaluation of a Smartphone Decision-Support Tool for Diarrheal Disease Management in a Resource-Limited Setting.	Bangladesh	Main district hospital and a sub-district hospital (1 0f 8) in the rural northern district of Nekrokona, a resource-limited area with a population of 2.2 million.	85 clinicians; 841 patients ≥2 months with diarrheal disease without comorbidities or severe malnutrition: 325 pre- and 516 post-intervention.	• 3 months long; • % Appropriate antibiotic prescription.
Pre- and Post-intervention Studies					
Bhullar, H.S. et al. (2016) [37]	Antimicrobial Justification form for Restricting Antibiotic Use in a Paediatric Intensive Care Unit.	India	14-bed paediatric intensive care unit in Children’s Hospital, Hyderabad.	1693 paediatric patients: 872 pre- and 821 post-intervention. Illness not reported.	• 21 months long; • % Receiving restricted antibiotics (RA).
Dehn Lunn, A. (2018) [39]	Reducing inappropriate antibiotic prescribing in upper respiratory tract infection in a primary care setting in Kolkata.	India	Outreach primary care clinics in rural Kolkata and West Bengal serving homeless and slum communities.	311 patients: 222 pre- and 92 post-intervention; 10 doctors, pharmacists, and other health workers. Illness: URTIs.	• 4 months long; • % Encounters with antibiotics.
Gray, A.Z. et al. (2015) [49]	Implementing WHO hospital guidelines improves quality of paediatric care in central hospital in Lao PDR.	Lao PDR	3 hospitals in Vientiane with a total 140-beds and a paediatric ICU in each hospital.	91 clinicians; 681 patients: 356 pre- and 325 post-intervention. Illness: pneumonia, diarrhoeal disease, LBW.	• 15 months long; • Mean Key CG indicator scores.
Hamilton, D. et al. (2018) [54]	Improving antimicrobial stewardship in the outpatient department of a district general hospital in Sierra Leone.	Serra Leone	Outpatient department of Medusa Hospital, a rural district general hospital.	128 patient’s baseline; 139 phase 1; 128 phase 2. ASP.	• 6 months long; • % Correct drug, dosage, duration.
Jaggi, N. et al. (2012) [40]	Control of multidrug resistant bacteria in a tertiary care hospital in India.	India	A 300-bed tertiary care private hospital in Gurgaon, Haryana.	28,971 clinical samples cultured. Illness: HAIs	• 36 months long; • DDD per 1000 bed-days.
Joshi, R.D. et al. (2019) [51]	Evaluation of a Hospital-Based Post-Prescription Review and Feedback Pilot in Kathmandu, Nepal.	Nepal	125-bed tertiary care hospital in Kathmandu Valley.	451 patients’ charts reviewed: 221 baseline and 230 post-intervention. ASP.	• 24 months long; • % Appropriate antibiotic therapy
Korom, R.R. et al. (2017) [47]	Brief educational interventions to improve performance on novel quality metrics in ambulatory settings in Kenya: a multi-site pre-post effectiveness trial.	Kenya	2 semi-urban primary health centres in Nairobi.	24 clinical officers; 475 charts of female patients aged 14 to 49 years. Illness: UTIs.	• 12 months long; • % Encounters with antibiotics; • % Appropriate antibiotic therapy.
Murni, I.K. et al. (2015) [45]	Reducing hospital-acquired infections and improving the rational use of antibiotics in a developing country: an effectiveness study.	Indonesia	Referral teaching hospital with 39-bed paediatric ward and 9-bed paediatric intensive care in Yogyakarta servicing 2.4 million people.	2646 paediatric inpatients with more than 48 hours hospital stay: 1227 pre- and 1419 post-intervention. Illness: HAIs.	• 26 months long; • Incidence of HAIs; • % Exposed to inappropriate antibiotics.
Patel, S. (2016) [41]	Impact of antibiotic stewardship strategy on the outcome of non-critical hospitalized children with suspected viral infection.	India	Paediatric ward of Shardaben Hospital, NHL Medical College, Ahmedabad.	1760 non-critical paediatric patients with suspected viral infections. Illness: suspected viral infection.	• 44 months long; • % RA used appropriately.
Singh, S. et al. (2019) [42]	Implementation and impact of an antimicrobial stewardship program at a tertiary care centre in South India.	India	Academic tertiary care referral hospital in Kerala with 1300 beds and 254 beds across 13 ICUs.	4613 patients received at least 1 antibiotic during inpatient stay. ASP.	• 23 months long; • Mean length of inpatient days;
Siddiqui, S. et al. (2007) [53]	Impact of antibiotic restriction on broad spectrum antibiotic usage in the ICU of a developing country	Pakistan	Tertiary care teaching hospital with 12-bed ICU in Karachi.	Sample size not reported. Illness: HAIs.	• 6 months long; • % RA prescribed as per CG; • % Cost RA • Mortality per 1000 inpatients.
Tamer, S. et al. (2015) [35]	Antimicrobial stewardship to optimize the use of antimicrobials for surgical prophylaxis in Egypt: a multicentre pilot intervention study.	Egypt	Five tertiary acute care surgical hospitals with infection control programs and ASPs teams.	1303 patients: 745 patients pre- and 558 post-intervention: ASP.	• 12 months long; • % Correct dose, duration, timing of antibiotics.
Tillekeratne, L.G. et al. (2015) [55]	Use of rapid influenza testing to reduce antibiotic prescriptions among outpatients with influenza-like illness in Southern Sri Lanka.	Sri Lanka	Outpatient department in a 1500-bed teaching hospital in Karapitiya with >1000 patients daily.	10 clinicians and 571 outpatients ≥ 1 year: 316 in phase 1 and 241 in phase 2. Illness: influenza-like illness.	• 20 months long; • % Encounters with antibiotics; • % Appropriate prescription.

ALMANACH = algorithms for the management of acute childhood illnesses; ARTIs = acute respiratory tract infections; ASP = antimicrobial stewardship program; CA = community acquired; CG = clinical guideline; DDD = defined daily dose; e-POCT = electronic-point of care tool; HA = hospital acquired; HAIs = healthcare associated infections; ICU = intensive care unit; LBW = low birth weight; LLMICs = low- and lower-middle-income countries; No. = number; PAL = practical approach to lung health; RA = restricted antibiotics; SSIs = surgical site infections; STIs = sexually transmitted diseases; URTIs = upper respiratory tract infections; UTIs = urinary tract infections.

**Table 2:** Interventions used in the studies for implementing and promoting adherence to antibiotic guidelines in LLMICs.

Intervention Activities		Aiken, AM et al. (2013) [46]	Akter, SF et al. (2009) [31]	Awad, AI et al. (2006) [56]	Bernasconi, A et al. (2018) [30]	Bhuller, HS et al. (2016) [37]	Chalker, J (2001) [59]	Chandy, SJ et al. (2014) [38]	Chowdhury, AK et al. (2007) [32]	Dehn-Lunn, A et al. (2018) [39]	Do, NTT et al. (2016) [60]	Gebretekle, GB et al. (2020) [36]	Gray, AZ et al. (2016) [49]	Hadi, U et al. (2008) [44]	Hamilton, D et al. (2018) [54]	Haque, F et al. (2017) [33]	Hoa, NQ et al. (2017) [61]	Jaggi, N et al. (2012) [40]	Joshi, RD et al. (2019) [51]	Keitel, K et al. (2017) [57]	Korom, RR et al. (2017) [47]	Murni, IK et al. (2015) [45]	Opondo, C et al. (2011) [48]	Petal, S et al. (2016) [41]	Sarma, H et al. (2019) [34]	Shao, AF et al. (2016) [58]	Shrestha, N et al. (2006) [52]	Singh, S et al. (2019) [42]	Siddiqui, S et al. (2007) [53]	Tamer, S et al. (2015) [35]	Tillekeratne, L et al. (2015) [55]	Trap, B et al. (2001) [62]	Wahstrom, R et al. (2003) [50]	Wattal, C et al. (2017) [43]
**CG origin**	**Antibiotic Guideline or Policy**	D	U	U	A	D	C	D	C	D	A	D	C	D	C	C	A	U	A	A	D	C	A	U	D	A	A	A	D	C	U	U	D	U
**Organisational**	**Management Endorsement**									X				X																				
	**Stakeholder Consensus**		X				X			X				X																				
	**Champions**											X																		X				
	**Institution Incentives**						X							X																				
	**AMS Programme**											X										X		X				X		X				
**Capacity buidling**	**WORKSHOPS & Seminars**		X	X			X			X			X	X		X	X				X	X			X	X				X	X			
	**Follow-up Training**						X							X																				
	**Academic Detailing**			X																	X													
	**Focus Group Discussion**									X			X								X	X											X	
**Monitoring & review**	**Audit & Feedback**			X						X		X	X								X	X						X		X			X	
	**Antimicrobial Restriction**					X																		X					X					
	**Reminders**			X							X											X												
	**Practice Supervision & Feedback**						X									X										X								
**CDSS**	**Quick Reference**												X				X					X			X					X				
	**Clinical Algorithms**				X															X						X								
	**Rapid Diagnostic Testing Tools**										X					X				X											X			
**Persua-Sive Activities**	**Sharing audit results across depts.**						X																											
	**Conditional Donation of Equipment and Funds**						X																											
**Result**	**Studies Showing the Intervention Had a Positive Direction of Effect**																																	

AMS = antimicrobial stewardship; CG = clinical guideline; CDSS = clinical decision support systems; Antibiotic guideline/policy: A = adapted; C = adopted; D = developed from scratch; U = unclear; X = strategies used singularly or in multifaceted interventions resulting in a measure of improvement in CG adherence.

**Table 3:** Effect direction plot summarising direction of effects of intervention outcomes of strategies used for implementing and promoting adherence to antibiotic guidelines in LLMICs.

Adherence to Clinical Guidelines Indicated by Improvement in Domain Areas						
Study	Study Design (Risk of Bias)	Reduction in Encounters with an Antibiotic	Antibiotics Prescribed Appropriate: Dose, Timing, Duration	Reduction in Defined Daily Doses (DDDs) Per 100/1000 Bed-Days	Reduction in Rate of Clinical Failure	Clinical Guideline Performance Indicator Scores
Keitel, K et al. (2017) [57]	RCT (L)	▲			▲	
Do, NTT et al. (2016) [60]	RCT (L)	▲				
Shoa, AF et al. (2017) [58]	RCT (L)	▲			▲	
Hoa, NQ et al. (2017) [61]	CRCT (L)		▼			▲
Tillekeratne, L et al. (2015) [55]	PPI (L)	▲				
Wahlstrom, R et al. (2003) [50]	RCT (M)					▲^
Awad, AI et al. (2006) [56]	CRCT (M)	▲				
Opondo, C et al. (2011) [48]	CRCT (M)		◄►			
Gebretekle, GB et al. (2020) [36]	QE (M)	▲				
Sarma, H et al. (2019) [34]	QE (M)		▲^			
Chandy, SJ (2014) [37}	ITS (M)			◄►		
Hadi, U et al. (2008) [44]	ITS (M)			▲^		
Wattal, C et al. (2017) [43]	ITS (M)			◄►		
Bernasconi, A et al. (2018) [30]	CPPI (M)	▲	▲			
Haque, F et al. (2017) [33]	CPPI (M)		▲			
Bhuller, HS et al. (2016) [37]	PPI (M)	▲				
Joshi, RD et al. (2019) [51]	PPI (M)	◄►				
Murni, IK et al. (2015) [45]	PPI (M)		▲			
Tamar, S et al. (2015) [35]	PPI (M)	▲	▲			
**§Two-tailed Sign Test: †L & M risk of bias**		***p* = 0.0027**	***p* = 0.1024**			
Trap, B et al. (2001) [62]	RCT (H)			◄►		
Chowdhury, AK et al. (2007) [32]	CRCT (H)	◄►				
Shrestha, N et al. (2006) [52]	CRCT (H)	◄►				◄►
Aiken, AM et al. (2013) [46]	ITS (H)		◄►			
Chalker, J et al. (2001) [59]	ITS (H)	▲	▲			
Akter, SF et al. (2009) [31]	CPPI (H)		▲			
Dehn Lunn, A et al. (2018) [39]	PPI (H)	▲^				
Gray AZ et al. (2015) [49]	PPI (H)					▲
Hamilton, D et al. (2018) [54]	PPI (H)		◄►			
Jaggi, N et al. (2012) [40]	PPI (H)	◄►		◄►		
Korom, RR et al. (2017) [47]	PPI (H)		▲			
Patel, S et al. (2016) [41]	PPI (H)					
Siddique, S et al. (2007) [53]	PPI (H)	▲	▲^			
Singh, S et al. (2019) [42]	PPI (H)		▲	▲	▲	
§Two-tailed Sign Test: for all studies		*p* = 0.0005	*p* = 0.0066			

Study design: RCT: randomised controlled trial; CRCT: cluster RCT; QR; quasi experimental; ITS: interrupted time series; CPPI: controlled pre- post-intervention; PPI: pre- post-intervention. Effect direction: upward arrow ▲ = positive impact, downward arrow ▼= negative impact, sideways arrow ◄► = no change/mixed effects. Sample size in intervention group: large arrow ▲ > 300; medium arrow ▲ > 100 to 300; arrow with hat ▲**^** ≤ 100. Study quality denoted by row colour: green = low risk of bias; amber = medium risk; red = high risk of bias. §Two tailed sign test *p* value with hypothetical probability of success in each subject being 0.5 calculated for domains with sufficient studies to do so (<5). † L = low and M = medium risk of bias.

## Data Availability

All additional data can be found in the supplementary files. There is no other data involved in this study.

## Supplementary Materials

The following are available online at https://www.mdpi.com/article/10.3390/tropicalmed6030166/s1, Table S1: PubMed search strategies used to identify studies investigating strategies for implementing and promoting antibiotic guidelines in LLMICs; Box S1: Description of the intervention strategies used in studies for implementing and promoting antibiotic guidelines in LLMICs; Table S2: (a) Results of risk of bias assessment of studies investigating strategies for implementing and promoting antibiotic guidelines in LLMICs; (b) Risk of bias assessment of interrupted time series studies investigating strategies for implementing and promoting antibiotic guidelines in LLMICs [24]. Figure S1: Bubble plot showing the variation in study design, research quality and number of intervention strategies implemented across studies investigating strategies for implementing and promoting antibiotic guidelines in LLMICs; Table S3: (a): Interventions, outcome measures and effect sizes for studies investigating strategies for implementing and promoting antibiotic guidelines in LLMICs; (b): Interventions, data summaries, outcome measures and trend changes reported in ITS studies; Table S4: PRISMA 2009 Checklist [21].
