# Supplementary material for: Strategies Used for Implementing and Promoting Adherence to Antibiotic Guidelines in Low- and Lower-Middle-Income Countries: A Systematic Review
Source: Trop Med Infect Dis. 2021 Sep 13;6(3):166. doi: 10.3390/tropicalmed6030166 (PMC8482147; doi:10.3390/tropicalmed6030166)
Supplement: Supplementary file 1 [file tropicalmed-06-00166-s001.zip › tropicalmed-1353597-supplementary.pdf]

Tropical Medicine and Infectious Disease

MDPI

Systematic Review

Strategies Used for Implementing and Promoting Adherence to Antibiotic Guidelines in Low- and Lower-Income Countries: A Systematic Review

Nicola D. Foxlee [1,*], Nicola Townell [2], Claire Heney [3], Lachlan McIver [4] and Colleen L. Lau [5]

[1] Department of Global Health, Research School of Population Health, Australian National University, Canberra, ACT 2600, Australia;
[2] Infectious Disease Department, Sunshine Coast University Hospital, Birtinya 4575, Queensland, Australia; nikkitownell@hotmail.com
[3] Pathology Queensland, Central Microbiology, Brisbane 4006, Queensland, Australia; claire.heney@health.qld.gov.au
[4] Rocketship Pacific Ltd., Port Melbourne, Melbourne 3207, Australia; lachlan@rocket-ship.org
[5] School of Public Health, University of Queensland, Herston 4006, Queensland, Australia and Research School of Population Health, Australian National University, Canberra, ACT 2600, Australia; colleen.lau@uq.edu.au
* Correspondence: nicola.foxlee@anu.edu.au; Tel.: +61-7-435-549-071

Supplementary Materials

Table S1. PubMed search strategies used to identify studies investigating strategies for implementing and promoting antibiotic guidelines in LLMICs.

**Search 1**	
LLMICs	Afghanistan OR Benin OR Burkina OR Faso OR Burundi OR "Central African Republic" OR Chad OR Comoros OR Congo OR Ethiopia OR Eritrea OR Gambia OR Guinea OR Bissau OR Haiti OR Liberia OR Madagascar OR Malawi OR Mali OR Mozambique OR Nepal OR Niger OR Rwanda OR "Sierra Leone" OR Somalia OR Sudan OR Syria OR Tajikistan OR Tanzania OR Togo OR Uganda OR Yemen OR Zimbabwe OR Angola OR Bangladesh OR Bhutan OR Bolivia OR "Cabo Verde" OR Cambodia OR Cameroon OR "Cote d'Ivoire" OR Djibouti OR Egypt OR "El Salvador" OR Georgia OR Ghana OR Honduras OR India OR Indonesia OR Kenya OR Kiribati OR Kosovo OR Kyrgyz OR Lao OR Lesotho OR Mauritania OR Micronesia OR FSM OR Moldova OR Mongolia OR Morocco OR Myanmar OR Nigeria OR Pakistan OR PNG OR "Papua New Guinea" OR Philippines OR "Sao Tome and Principe" OR "Solomon Islands" OR "Sri Lanka" OR Swaziland OR "Timor Leste" OR Tunisia OR Ukraine OR Uzbekistan OR Vanuatu OR Zambia OR Developing countries [Mesh]
AND	Clinical Protocols"[Mesh] OR "Practice Guidelines as Topic"[Mesh] OR "clinical protocols"[tw] OR "practice guidelines"[tiab] OR "clinical practice guidelines"[tw] OR "therapeutic guidelines"[tw] OR guidelines[tw] OR "antibiotic policy" OR protocol[tw]
AND	Implement* OR promot* OR disseminat* OR uptake OR adopt* OR adhere* OR compl*
AND	Antibiotic OR antimicrobial OR antibacterial OR "Anti-Bacterial Agents/therapeutic use"[Mesh]
**Search 2**	
LLMICs	As above
AND	Clinical Protocols"[Mesh] OR "Practice Guidelines as Topic"[Mesh] OR "clinical protocols"[tw] OR "practice guidelines"[tiab] OR "clinical practice guidelines"[tw] OR "therapeutic guidelines"[tw] OR guidelines[tw] OR "antibiotic policy" OR protocol[tw]
AND	strateg*[tw] OR intervent*[tw] OR approach[tw] OR programme[tw] OR scheme[tw] OR stewardship[tw] OR education* OR workshop[tw] OR feedback[tw] OR audit[tw] OR pharmacist[tw] OR restrict* OR authorisation[tw]
AND	Antibiotic OR antimicrobial OR antibacterial OR "Anti-Bacterial Agents/therapeutic use"[Mesh]
**Search 3**	
LLMICs	As above
AND	Clinical Protocols"[Mesh] OR "Practice Guidelines as Topic"[Mesh] OR "clinical protocols"[tw] OR "practice guidelines"[tiab] OR "clinical practice guidelines"[tw] OR "therapeutic guidelines"[tw] OR guidelines[tw] OR "antibiotic policy" OR protocol[tw]
AND	educat*[tw] OR seminarltw] OR audit*[tw] OR feedback[tw] OR monitor[tw] OR "opinion leader"[tw] OR assessment[tw] OR peer-review[tw] OR multifaceted[tw] OR reminders[tw] smartphone[tw] OR m-health[tw] OR "mobile technology"[tw] OR telemedicine [Mesh] OR workshop* OR order form OR antibiotic restriction
AND	Antibiotic OR antimicrobial OR antibacterial OR "Anti-Bacterial Agents/therapeutic use"[Mesh]
**Search 4**	
LLMICs	As above
AND/OR	("Practice Guidelines as Topic"[Mesh] OR "clinical guideline" OR "practice guideline" OR "Clinical Protocols"[Mesh] OR protocol) AND ("Health Plan Implementation/methods"[Mesh] OR implement* OR promot* OR disseminat* OR compliance OR comply OR adher*)
AND	Antibiotic OR antimicrobial OR antibacterial OR "Anti-Bacterial Agents/therapeutic use"[Mesh]
**Search 5**	
LLMICs	As above

AND	"Clinical Protocols" [Mesh] OR "Practice Guidelines as Topic"[Mesh] OR "clinical protocols"[tw] OR "practice guidelines"[tiab] OR "clinical practice guidelines"[tw] OR "therapeutic guidelines" OR guidelines[tw] OR "antibiotic policy" OR protocol[tw] OR "standard treatment guideline"
AND	"Communicable Diseases"[Mesh] OR "infectious disease"[tw] OR "bacterial infection"[tw] OR "bacterial infections"[tw]
AND	Antibiotic OR antimicrobial OR antibacterial OR "Anti-Bacterial Agents/therapeutic use"[Mesh]

Mesh = medical subject heading; tiab = title, index, abstract; tw = text word.

Box S1. Description of the intervention strategies used in studies for implementing and promoting antibiotic guidelines in LLMICs.

Organisational

1. **Endorsement**: the guideline or policy has been approved and is supported by the Organisation. It may be a compulsory tool.
2. **Consensus**: all staff agree on the guideline or policy recommendations thereby increasing their willingness to implement them.
3. **Champions:** people within the organisation who strongly support the use of the guideline or policy, advocate and promote its acceptance to management and work colleagues and who are instrumental in its implementation.
4. **Incentives**: free pathology testing, donation of equipment, funds to purchase antimicrobials.
5. **Antimicrobial stewardship programs (AMS)**: activities which focus on optimising prescribing practices, infection, prevention and control and quality of care and include guideline implementation.

Capacity Building

1. **Workshops and seminars**: delivered by lecture or interactive discussion or case-based-learning on antimicrobial resistance, optimal prescribing and use of guidelines.
2. **Refresher training**: follow up training sessions on use of the guideline/policy or testing tools.
3. **Academic detailing:** face-to-face education provided to prescribers by specially trained health professionals (commonly a pharmacist) with the aim of improving prescribing practices.
4. **Focus group discussions:** lunchtime meetings to discuss ways to improve prescribing behaviours, barriers to optimal prescribing and difficult cases and facilitated by a senior clinician or a supervisor.

Monitoring and Review

1. **Audit and feedback:** generally conducted monthly and results followed-up with recommendations for improvement delivered in print or face-to-face or with focus group discussions to identify and overcome barriers to guideline adherence.
2. **Antimicrobial restriction:** requires completion of a justification form and consultation with pharmacist, infectious diseases clinician or senior clinician within a certain timeframe.
3. **Reminders:** follow-up instructions in print, email or face-to-face.
4. **Supervision:** conducted by pharmacists or other senior health professionals at regular intervals and maybe combined with face-to-face discussions based on the results.

Clinical Decision Support Systems (CDSS)

1. **Quick reference (job aids)**: wall charts, posters, leaflets, booklets, and drug lists in various formats: print, on mobile devices, hospital intranet, department computers.

2. **Clinical algorithm**: flow charts displaying a sequence of clinical decisions to assist health care professionals in the diagnosis and management of medical problems.
3. **Rapid diagnostic testing tools:** applications, such as point of care tests which analyse medical data to assist healthcare providers to make clinical decisions at the point of care.

Persuasive strategies

1. **Sharing audit results and/or prescribing pattern results** with other departments or staff for the purpose of influencing the actions of one group or the other.
2. **Providing incentives:** donation of equipment and funds based on showing improvement in prescribing.

Table S2. (**a**) Results of risk of bias assessment of studies investigating strategies for implementing and promoting antibiotic guidelines in LLMICs.

Study Type	RCT					CRCT					QE		CPPI					PPI											
Author ID / Quality criteria (Total points)	Keitel, K et al (2017) [56]	Do, NTT et al (2016) [59]	Trap, B et al (2001) [61]	Shao, AF et al (2015) [57]	Wahlstrom, R et al [illegible]	Awad, AI et al (2006) [55]	Chowdhurry, AK et al [illegible]	Hoa, NQ et al (2017) [60]	Opondo, C et al (2011) [illegible]	Shrestha, N et al (2007) [illegible]	Gebretekle, GB (2020) [illegible]	Sarma, H et al (2019) [33]	Akter, SFU et al (2009) [illegible]	Bernasconi, A et al (2018) [illegible]	Haque, F et al (2017) [32]	Bhuller, HS et al (2016) [illegible]	Dehn Lunn, A (2018) [38]	Gray, AZ et al (2015) [48]	Hamilton, D et al (2018) [illegible]	Haque, F et al (2017) [32]	Jaggi, N et al (2012) [39]	Joshi, RD et al (2019) [50]	Korom, RR et al (2017) [illegible]	Murni, IK et al (2015) [44]	Patel, S et al (2016) [40]	Siddiqui, S et al (2007) [illegible]	Singh, S et al (2019) [41]	Tamar, S et al (2015) [34]	Tillekeratne, L et al (2015) [illegible]
Reporting (10)	L	L	M	L	L	L	H	L	M	H	M	M	M	L	L	M	M	M	H	L	H	L	M	L	H	H	M	M	L
External validity (3)	L	L	H	L	H	M	H	L	H	H	L	H	L	H	M	M	H	H	M	M	M	M	H	L	H	H	H	L	L
Internal validity - bias (6)	L	L	M	L	M	M	H	M	H	H	M	M	M	L	M	M	H	M	M	M	H	M	M	M	H	H	H	M	L
Internal validity - confounding (7)	L	L	H	L	M	M	H	M	H	H	H	H	H	H	M	H	H	H	H	H	H	M	H	M	H	H	H	H	L
Power (2)	L	L	H	L	H	L	H	L	H	H	M	M	L	L	H	L	H	L	H	H	H	L	L	L	H	H	H	L	L
Final result (28)	Low	Low	High	Low	Medium	Medium	High	Low	Medium	High	Medium	Medium	Medium	Medium	Medium	Medium	High	High	High	Medium	High	Medium	Medium	Medium	High	High	High	Medium	Low

RCT = randomised control trial; CRCT = cluster RCT; QE = Quasi-experimental study; CPPI = Cluster pre- and post-intervention study; PPI = pre- and post-intervention study; L (yellow) = low risk of bias; M (brown) = medium risk of bias; H (red) = high risk of bias. Score = L = 22-28; M = 15-21; H = 0-14.

Table S2. (**b**) Results of risk of bias assessment of interrupted time series studies investigating strategies for implementing and promoting antibiotic guidelines in LLMICs.[23].

		**Interrupted Time Series Studies**				
	Study ID<br>Quality criteria<br>(Total points)	Aiken, A et al (2013) [45]	Chalker, J et al (2001) [58]	Chandy, SJ et al (2014) [37]	Hadi, U et al (2008) [43]	Wattal, C et al (2017) [42]
1	Intervention independent of other changes	M	M	M	L	M
2	Intervention unlikely to affect data collection	L	L	L	L	M
3	Primary outcome assessed blindly or measured objectively	H	L	H	L	L
4	Primary outcome reliable or measured objectively	M	M	M	L	M
5	Dataset at each timepoint covered ≥80% of participants	M	H	L	L	M
6	Shape of intervention effect pre-specified	H	H	L	H	M
7	Rationale for number and spacing of datapoints described	L	H	L	M	L
8	Study analysis was conducted appropriately	M	H	M	M	M
	Final Result	High	High	Medium	Medium	Medium

L (yellow) = low risk of bias; M (brown) = medium risk of bias; H (red) = high risk of bias.

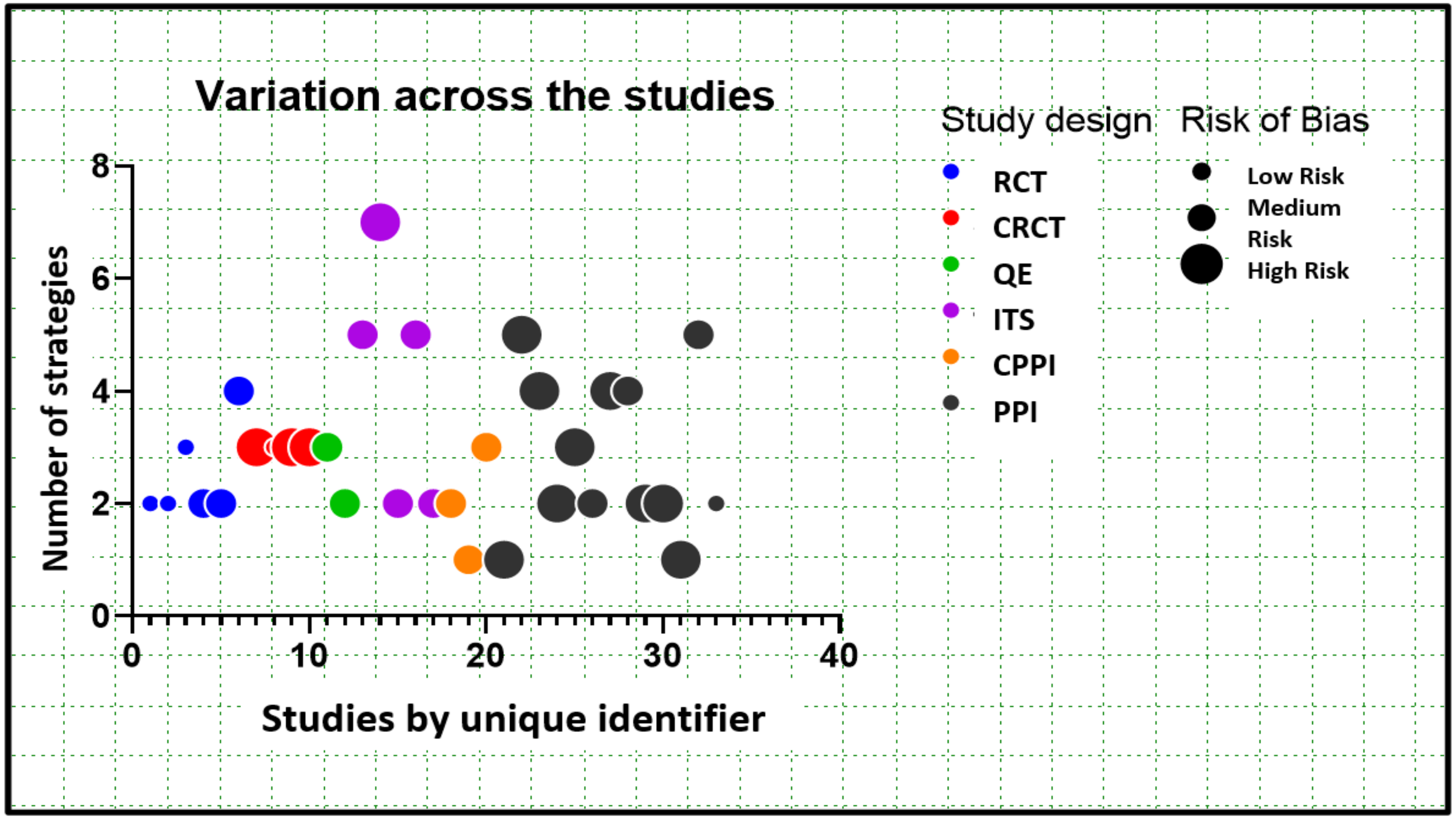

Figure S1. Bubble plot showing the variation in study design, research quality and number of intervention strategies implemented across studies investigating strategies for implementing and promoting antibiotic guidelines in LLMICs. Unique identifier = number 1 to 33; Study design: RCT = randomised controlled trial; CRCT = controlled RCT; QE = quasi experimental; ITS = interrupted time series; CPPI = controlled pre-post-intervention; PPI = pre-post-intervention

Table S3. (**a**) Interventions, outcome measures and effect sizes for studies investigating strategies for implementing and promoting antibiotic guidelines in LLMICs.

Citation (Year)	Intervention	Study Details Not Reported (nr)	Data Summary	Outcome Measure; Effect Size (95% CI), *P* value
**RCT**				
Keitel, K. et al. (2017) [56]	Intervention group: electronic ALMANACH algorithm with point of care tests (POCTs) available on an android-based mobile tool. The POCTs (oxygen saturation, heart rate, blood glucose, and Hb for detecting malaria infection, and identifying bacterial and viral diseases) were used to triage children with severe disease.<br>Control group: ALMANACH algorithm only.	• 13 months<br>• *N* = 3169<br>• Child<br>• age 24 to 59 months<br>• Severe infectious disease	1) Clinical failure by day 7 in e-POCT was 2.33% (37/1586) compared with ALMANACH at 4.1% (65/1583).<br>2) Antibiotic prescriptions on day 0 in e-POCT was 11.5% (182/1586) compared with ALMANACH reduction of 29.7% (470/1583).	1) RR<br>0.57 (0.38; 0.85)<br>*p* 0.005<br>2) RR<br>0.39 (0.33; 0.45)<br>*P* < 0.001
Do NTT et al. (2016) [59]	Intervention: patients with symptoms of ARI received point of care testing (C-reactive protein) and use of ALMANACH algorithm compared with Control: usual care and local treatment guideline.	• 15 months<br>• *N* = 2,037.<br>• Child and adult<br>• age 1 to 65 years<br>• ARIs	1)Proportion prescribed antibiotics within 14 days was 64% (581/902) in experimental group compared with 78% (738/947) in control.<br>2) Antibiotics in urine on days 3,4,5: 267/877 30% in experimental group; 314/882 36% in control.	1) OR (adjusted)<br>0.49 (0.40; 0.61) p<0.001<br>2) OR (adjusted)<br>0.78 (0.63; 0.95)<br>*p* 0.015
Shao, A.F. et al. (2016) [57]	Intervention: ALMANACH algorithm running on mobile technology with face-to-face training on e-tool and supervision during consultations.<br>Control: normal care.	• 7 months<br>• *N* = 1465<br>• Child<br>• age 24 to 59 months<br>• ARIs	1) Proportion prescribed antibiotics on day 0: experimental: 15.4% (130/842); compared with Control: 38.7.% (241/623);<br>2) Proportion cured by day 7. Experimental: 97.3% (815/842) compared with Control: 92% (573/623).	Absolute % difference<br>1) 68.9 (68.5; 69.2) *p* < 0.001<br>2) 5.3% (6.3; 4.3) *p* < 0.001
Trap, B. et al. (2001) [61]	Supervision by specially trained pharmacist on 1) stock management and 2) adherence to CGs compared with control group.	• 12 months<br>• *N* = 62<br>• Child and adult<br>• Non-bloody diarrhoea<br>• ARTI<br>• Genital ulcer<br>• Urethral discharge	CG adherence according to: correct drug, dose and duration (DDD) for: 1) non-bloody diarrhoea; 2) ARTI 3) urethral discharge in men; 4) genital ulcer disease. Intervention compared with control group increased for all infections:<br>1) 47% (*p* <0.001);<br>2) ARI 17% (*p*-value 0.001);<br>3) urethral discharge 32.5% (*p* 0.042);<br>4) genital ulcer 15.5% increase (*p* 0.20)<br>overall increased adherence 29% (*p* 0.34. (Stock management data not reported here.)	Absolute % difference<br>29% *p* 0.34

Wahlstrom, R. et al. (2003) [49]	Promotion of new clinical guideline (CG): Intervention: CG plus performance indicator review with monthly feedback and discussions on improving prescribing by specially trained facilitators. Control: CG only.	• 12 months<br>• N = 23,156<br>• Child and adult<br>• Malaria, diarrhoea, pneumonia	Overall changes in mean CG performance indicator score:<br>Diarrhoea: 0.86 (0.28; 1.42)<br>*p*-value 0.006.<br>Malaria: 0.65 (0.21; 1.13)<br>*p*-value 0.006.<br>Pneumonia: 0.35 (-0.52; 1.51) *p*-value 0.401.	Difference in mean CG indicator scores 0.63 (0.16; 1.12) p 0.012
**CRCT**				
Awad, A.I. et al. (2006) [55]	4 groups:<br>1) Audit and feedback.<br>2) audit and feedback plus 4-hour interactive educational seminar.<br>3) audit and feedback plus academic detailing.<br>4) control with usual care.	• 6 months<br>• N = 1800<br>• patient encounters	Mean difference in encounters with an antibiotic prescription a) 1-month b) 3-month post-intervention: experimental groups 1-3, control group 4:<br>1) reduced from a) 13.4 to 11.6; b) 13.4 to 10.8.<br>2) reduced from a) 14.2 to 8.6; b) 14.2 to 7.3.<br>3) reduced from a) 14.0 to 7.3; b) 14.0 to 6.6<br>4) control a)14.3 to 14.0 ; b)14.3 to 14.6	Mean difference in encounters<br>1a) 1.4 (0.4; 3.3)<br>p 0.121<br>1b) 2.8 (1.1; 4.6)<br>p 0.004<br>2a) 5.3 (3.4; 7.1)<br>$p < 0.001$<br>2b) 7.1 (5.4; 8.9) $p < 0.001$<br>3a) 6.3 (4.4; 8.2)<br>$p < 0.001$<br>3b) 7.7 (5.9; 9.5)<br>p <0.001<br>4) No change
Chowdhury, A.K. et al. (2007) [31]	3 groups:<br>1) CG with briefing; prescribing audit and feedback; focus group discussions on pneumonia prescribing;<br>2) CG only;<br>3) control group - no intervention.	• 7 months<br>• N = nr<br>• Child<br>• age < 5 years<br>• ARI<br>• Diarrhoea	Encounters with an antibiotic in ARI scored against CG:<br>1) Group 1. pre-intervention 90.3% and post- 66.6% with significant reduction in 6/8 health complexes;<br>2) Group 2. pre-intervention 85.9% and post - 70.7%;<br>3) Control Group reduction was 8.2%.<br>Mean number of drugs per encounter: group 1) 1.24; group 2) 1.2; group 3) 1.24. resulting in no change amongst groups.<br>Diarrhoea not shown.	Absolute % difference<br>1) 23.7%<br>2) 15.2%<br>3) 8.2%
Hoa, N.Q. et al. (2017) [60]	Multifaceted educational intervention: training sessions on antibiotic management for ARI; interactive case scenarios; management training; posters displaying ARI algorithms distributed to health facilities. Follow-up knowledge attitudes and prescribing (KAP) survey based on WHO IMCI guidelines to assess knowledge post-intervention. Control arm with usual care.	• 7 months<br>• N = 2021<br>• child<br>• age ≤ 5 years<br>• ARIs	1) Overall mean difference in ARI KAP scores for appropriate prescribing:<br>1.17 (pre- 4.7; post-intervention 5.87) and control arm: 0.48 (pre-4.49; post-intervention 4.97)<br>2) Appropriate prescribing: intervention arm pre- and post-intervention: 516 (81%) and 412 (64%)<br>control arm pre- and post-: 279 (73%) and 265 (74%)	Mean difference KAP scores<br>1) 0.69 p 0.054<br>2) OR<br>0.556 p <0.001

Opondo, C. et al. (2011) [47]	Intervention: 5days of CG training; quick reference guides; monthly supervision visits; face-to-face feedback on progress and 6 monthly survey results. Control: partial intervention: 1.5 days didactic training on use of CG, written feedback and job aids.	• 36 months<br>• $N = 4232$<br>• Child<br>• age 2 to 59 months<br>• Non-bloody diarrhoea	Proportion of children receiving antibiotics inappropriately at endpoint according to CG: Intervention 52.6% (313/594) compared with control hospitals 77.2% (437/566); Adjusted difference 32% (0.-06; 0.88) p-value 0.077.	OR (adjusted) 0.32 (0.06; 1.88) p 0.077
Shrestha, N. et al. (2006) [51]	WHO Practical Approach to Lung Health guidelines (PAL); prescriber consensus reached on CG use; 5 days guidelines training; quick reference material (examination forms and posters) disseminated.	• 12 months<br>• $N = 407$<br>• Child and adult<br>• Asthma<br>• COPD, pneumonia	Outcome measured using WHO Rational Use of Drugs indicators.<br>1) Encounters with an antibiotic;<br>2) Adherence to PAL CG<br>Neither 1 nor 2 was statistically significant.	1) OR (adjusted) 0.37<br>2) OR (adjusted) 2.29
**QES**				
Gebretekle, G.B. et al. (2019) [35]	Phase 1: development of institutional CG based on annual antibiogram and uploaded on online AMS platform.<br>Phase 2: 4-day interactive training on AMS, treatment for common syndromes, interpretation of laboratory results.<br>Phase 3: audit and feedback conducted by pharmacist.	• 15 months<br>• $N = 1264$<br>• child and adult	Antimicrobial utilization during and post-intervention as:<br>1) Treatment duration—number of consecutive days patient receives an antibiotic: 8.7 ± 6.9 intervention and 12.8 ± 11.7 post-intervention<br>2) Days of therapy (DOT per 1000 patient days): 754/1000 intervention and post-intervention 1549/1000 patient days representing two-fold increase at end of intervention.	Absolute mean difference<br>1) 4.1 (−0.7; 8.9)<br>p 0.002<br>Absolute increase in DOTs/1000 patient days<br>2) 795 (718.8; 870.4)
Sarma, H. et al. (2019) [33]	Provision of job aids, user-friendly instructions for prescribing and dispensing appropriate treatment using amoxicillin instructions (according to treatment regimen), and training for treatment of childhood pneumonia.	• 4 months<br>• $N = 94$<br>• adult<br>• pneumonia	Adherence behaviour to treatment regimen for pneumonia using appropriate:<br>dose for age, timing and duration (5 days): intervention group 18/56 (32%) compared with control group 1/38 (3%)	Absolute % difference 29%
**CPPI**				
Akter, S.F. et al. (2009) [30]	Introduction of consensus-based standard treatment guidelines (STGs) disseminated to all hospitals; Intervention hospitals: received 20 short interactive training sessions over 5 days targeted to all prescribers in paediatric wards.<br>Control hospitals: STGs only.	• 12 months<br>• $N = 3466$<br>• child<br>• pneumonia, diarrhoea	Proportion (specific number per group not reported) of cases with appropriate antibiotic treatment. Experimental:<br>(1): 66.7% pre-; 83.1% post-intervention for pneumonia.<br>(2) 28% pre- and 86.8% post-intervention for diarrhoea.<br>Control:<br>(1) 27% pre- and 30% post-intervention for pneumonia.<br>(2) 79.6 pre- and 81.8% post-intervention for diarrhoea.	Absolute % difference<br>1)<br>13.4% $p < 0.001$<br>Absolute % difference<br>2)<br>56.8%<br>p 0.002

Bernasconi, A. et al. (2018) [29]	CDSS: an algorithm running on mobile technology adapted from WHO's ALMANACH with standard treatment guidelines.<br>Intervention group: received instruction on tool.<br>Control group: routine care.	• 16 months<br>• *N* = 8622<br>• child<br>• age 2 months to ≤5 years	1) proportion receiving at least 1 antibiotic baseline: 63.8% 367/575: and post-intervention 21.83% (1756/8047);<br>2) adherence to guideline: baseline 38.1% (219/575) and post-intervention 100% (575/575)	Absolute % difference 1)<br>0.42 (0.37; 0.46)<br>$p < 0.001$<br>Absolute % difference 2)<br>0.62% (0.57; 0.65) $p < 0.001$
Haque, F. et al. (2017) [32]	Intervention: CDSS: smartphone adaptation of the ALMANACH algorithm for the management of diarrheal diseases and a rehydration calculator for IV fluid ordering by rehydration status and volume recommendation.<br>Control: usual care.	• 3 months<br>• N = 841<br>• child<br>• age 11 to 17 years and adult<br>• diarrhoeal diseases	CG adherence based on appropriate prescription of azithromycin<br>(1a) District level pre- and during intervention:13%; 25/52 compared with 87% 334/384;<br>(1b) subdistrict level: 63% 48/76 compared with 99% 84/85.<br>(2a) District level: pre- and post-intervention 77% compared with 41%<br>(2b) Sub-district: 99% compared with 23%	1a)<br>RR 6.9 p <0.001<br>1b)<br>RR 1.6 p 0.35<br>2a)<br>RR 0.5 $p < 0.001$<br>2b)<br>RR 0.2 $p < 0.001$
**PPI**				
Bhuller, H.S. et al. (2016) [36]	Antibiotic restriction policy with justification form and list of restricted antimicrobials: to be completed within 24hrs, cultures requested, consultant notified within 48-72hrs; ID consultant to OK treatment continuation. Reviewed day 7 continuation requiring a repeat of the process.	• 21 months<br>• *N* = 1693<br>• Child	Proportion receiving restricted antibiotics:<br>Pre-intervention 40.5% (353/872) received at least 1 of the restricted antibiotics.<br>Post-intervention cohort 34.6% (284/821) received at least 1 of the restricted antibiotics.	Absolute % difference<br>5.9% (0.12; 0.105)<br>p 0.0122
Dehn Lunn, .A. et al. (2018) [38]	CG development and expanded coding for URTI; repeated process of audit and feedback following Plan, Do Study Act (PDSA) cycles:<br>Cycle 1: interactive educational seminar sessions on prescribing and Cycle 2: one-to-one case-based discussion.	• 4 months<br>• *N* = 222<br>• Child and adult<br>• URTIs	Reducing encounters with antibiotics according to PDSA cycles.<br>Baseline: 62.6% (139/222) patient encounters resulted in at least one antibiotic;<br>Post-cycle 1: 52.2% (48/92) encounters resulted in an antibiotic;<br>Post-cycle 2: encounters with an antibiotic had declined to 7.8% (5/69) of patient encounters	Absolute % difference<br>55.4%
Gray, A.Z. et al. (2016) [48]	WHO Pocketbook of hospital care for children adapted for Lao use; stakeholder consensus; opinion leaders; dissemination of pocketbook to all staff; small group interactive case-based workshops focussing on pocketbook chapters; audit and feedback.	• 15 months<br>• *N* = 3610<br>• Child<br>• age <28 days to <15 years	CG performance based on key indicators: 1) pneumonia: pre-intervention 485/900; post-intervention 690/900.<br>2): diarrhoea: pre-intervention 589/1080; post-intervention 741/1080.	Absolute mean difference<br>1) 22.78% (18; 27)<br>$p < 0.001$<br>2) 14.08% (18; 10)

		• diarrhoea, pneumonia, low birth weight	3) Low birth weight pre-intervention 856/1530; post-intervention 1163/1530.	$p < 0.001$<br><br>3) 20.07% (16.7; 23.2) p < 0.001
Hamilton, D. et al. (2018) [53]	Antimicrobial stewardship programme: CG adapted from international CG sources; PDSA cycles.<br>Cycle 1: CG introduced and explained to all clinicians followed by implementation; each clinician's patient records audited and followed-up by one-on-one feedback;<br>Cycle 2:	• 6 months<br>• *N* = 610<br>• Child and adult	Appropriate antibiotic prescribing with correct drug, duration and dose according to CG – outpatient records reviewed.<br>Baseline 161/243 (66%) appropriate choice and 86/251 (35%) correct drug and duration;<br>Post-cycle 1: 241/283 (85%) appropriate choice and 151/283 (53%) correct drug and duration<br>Post-cycle 2: 126/194 (65%) appropriate choice and 84/194 (43%) correct drug and duration	Absolute % difference 7.8 % (0.1; 0.8)
Jaggi, N. et al. (2012) [39]	ASP on AMR. 2 phased intervention:<br>Baseline patterns of antibiotic resistance in gram-negative isolates were reviewed.<br>1) Development and implementation of CG and infection control policy; and training in infection and prevention control procedures and practices and AMR<br>32) Formation and functioning of AMS Committee	• 36 months<br>• *N* = 5615<br>• positive culture	Monthly antibiotic consumption (DDDs per 1000 inpatient days) calculated according to WHO Antibiotic Therapeutic Classification system (ATC) for 2009.<br>Baseline: ESBLs (*E.coli* and *K. pneumoniae*): 55.3%.<br>Phase 1: 4.7% reduction in ESBLs (*E.coli* and *K. pneumoniae*) and 40.8% in carbapenem-resistant pseudomonas;<br>Phase 2: further 24.7% reduction in carbapenem-resistant Pseudomonas Overall 65.5% reduction in CR-Pseudomonas.<br>Rates of ESBLs in *E. coli* and *K. pneumoniae* fluctuated with no clear change.	Absolute % difference Mixed results
Joshi, R.D. (2019) [50]	Development of CGs; CG training and AMR training; audit of patient charts and physician logbooks and feedback conducted by physicians engaged as champions.	• 24 months<br>• *N* = 451<br>• child and adult<br>• medicine, surgery, obstetrics and gynaecology wards	Overall proportion of prescriptions appropriately:<br>a) justified, b) de-escalated, documented, c) CG followed in first 72 hrs, d) CG followed for definitive therapy e) documented correctly pre-compared with post-intervention:<br>1) Medicine—improved<br>2) Surgery—below baseline (2.75%)<br>3) Obstetrics and Gynaecology—below baseline (9%)	Absolute % difference<br>1)<br>27% (20.82; 32.87)<br>2)<br>−2.75% (−3.4; −8.9)<br>3)<br>−9.08% (−0.65; −18.6)
Korom, R.R. (2017) [46]	Development of CG for uncomplicated UTI; workshops to introduce guideline and discuss the rationale; interactive peer-to-peer review of patient documentation pertaining to guideline with feedback	• 12 months<br>• *N* = 475<br>• females<br>• age 14 to 49 years	Proportion of encounters with appropriate antibiotic therapy: baseline: 19% compared with 68% post-intervention. Total patient records reviewed: 475;<br>Number per group pre- and post-intervention not provided.	Absolute % difference 49%

	on compliance and discussion on ways to improve; open discussion on examination of a Kenyan study about AMR in uropathogens.	• uncomplicated UTIs		
Murni, I.K. et al. (2015) [44]	ALMANACH and hand hygiene campaign; educational seminars on rational prescribing based on the guidelines; reminders (checklists); audit and feedback at ward meetings.	• 26 months<br>• *N* = 2646<br>• child with<br>• ≥48 hours hospital stay	1) Incidence of HAIs: baseline 22.6% (277/1227) ; post-intervention 8.6% (123/1419)<br>2) Proportion of patients exposed to inappropriate antibiotic treatment baseline: 43% (336/780) and 20.6% (182/882) post-intervention.	1) RR 0.46 (0.40; 0.55) $p < 0.001$<br>2) RR 0.38 (0.31; 0.46) $p < 0.001$
Patel, S. et al. (2016) [40]	Antimicrobial stewardship programme:<br>CG developed;<br>Antibiotic restriction policy requiring prior approval for restricted antibiotics;<br>Justification forms to be completed and approved by ASP committee to commence antibiotic therapy.	• 44 months<br>• *N* = 1,760<br>• Child<br>• Age 3 months to 12 years<br>• Non-febrile fever	3143 patients hospitalised in the 4 years with 1760 (56%) with suspected viral infection not requiring antibiotics.<br>Rate of usage was 3.9 vials per patient in 2011–2013 (10545 vials/ 2779 admissions) compared with 2.36 vials per patient 2014–2015 (7446 vials/3143 admissions) showing 40% reduction over 4 years	Absolute % difference 40%
Siddiqui, S. et al. (2007) [52]	Antimicrobial stewardship programme:<br>Antibiotic restriction policy developed based on broad spectrum antibiotic usage in ICU limited to 72 hrs.<br>Cultures to be obtained prior to therapy;<br>Approval required from ID clinician or pharmacist.	• 6 months<br>• *N* = not reported<br>• 12-bed ICU	Change in defined daily doses per 1000 bed-days pre- and post-intervention:<br>33% (p value 0.04) (2.67 – 2.09)<br>Overall reduction in broad spectrum antibiotics 34% (498/1288)<br>Compliance with CG was 89%	Absolute % difference 33% *p* 0.04
Singh, S. et al. (2019) [41]	Antimicrobial stewardship programme (ASP):<br>ASP committee;<br>CG developed internally based on local antibiogram;<br>Justification forms for restricted antibiotics;<br>post-prescriptive audit and feedback.	• 23 months<br>• *N* = 48,555<br>• ICU<br>• AMS	!) Average length of stay decreased from 6.6 days to 6.4 days;<br>2) mortality per 1000 inpatients improved from 31.6% to 28.9%.<br>3) Impact ASP on cost of consumption - mean monthly cost of restricted drugs dropped by 14.4% compared to pre-intervention (p-value 0.03).<br>4) Adherence to CG for prescribing restricted drugs: 41% (201/490) pre-and 54% (318/584) post-intervention<br>5) Total patient days pre-intervention = 308,040 and post-intervention = 311,640	Absolute % difference<br>1) 3%<br>2) 2.7%<br>3) 14.4% *p* 0.03<br>4) 13%<br>5) 1.2%
Tamar, S. et al. (2015) [34]	AMS programme: IPC committee leadership; surgical prophylactic policy adopted; surgeon champions; 2-day training for all relevant staff; on-the-job training for juniors and residents; audit and feedback twice weekly.	• 12 months<br>• *N* = 1303<br>• child and adult<br>• obs. and gyn. orthopaedics gen. surgery	1) Proportion of patients who received at least one antibiotic dose within 60 minutes before incision: baseline: 283/745 (37.6%) and post-intervention: 232/558 (41.6%)<br>2) Optimal post-operative duration (≤24hrs after surgery): baseline 22/745 (3%) post-intervention:160/558 (29%).	Absolute % difference 1) 4% (0.09; 0.01)<br>Absolute % difference 2) 26% (0.29; 0.22) $p < 0.001$

Tillekeratne, L. (2015) [54]	Phase 1: routine care delivered in OPD with Rapid Antigen-based Test (RAT) for influenza A and B used for surveillance only, results withheld from clinician; clinician receives information session about RAT ; phase 2: results of RAT released to patient and clinician after treatment given; phase 3: questionnaire for patients 1-4 weeks post OP visit.	• 20 months<br>• *N* = 571<br>• child and adult<br>• outpatient<br>• ARTIs	1) Proportion of all outpatients receiving an antibiotic at baseline 81.3% (256/316) compared with 69.3% (167/241) post-intervention.<br>2) Proportion of influenza-like illness patients receiving an antibiotic at baseline: 83.7% (264/316) compared with 62.3% (150/241) post-intervention.	Absolute % difference<br>1) 12% (0.04; 0.19) $p < 0.001$<br>2) 17.9% (0.09; 0.25) $p < 0.001$

AMS = antimicrobial stewardship; ASP = antimicrobial stewardship programme; ALMANACH = algorithms for the management of acute childhood. illnesses; ARTIs = acute respiratory tract infections; CA = community acquired; CG = clinical guideline; CDSS = clinical decision support systems; e-POCT= electronic-point of care tool; HA = hospital acquired; HAIs = healthcare associated infections; ICU = intensive care unit; IPC = infection prevention and control; OPD = outpatient department; SSI = surgical site infections; STIs = sexually transmitted diseases; URTIs = upper respiratory tract infections; UTIs = urinary tract infections.

Table S3. (**b**) Interventions, data summaries, outcome measures and trend changes reported in ITS studies.

Citation (Year)	ITS Studies	Study Size and No. Data Points	Data Summary	Outcome Measure	Trend Change
Aiken, A.M. et al. (2013) [45]	Surgical prophylaxis policy endorsed by management; staff consensus obtained; training workshops and seminars on policy; audit and feedback; supervision; job aids, wall charts and patient posters.	• *N* = 3343<br>• 66	a) Pre-intervention: no evidence of monthly trend changes in risk of SSIs with 6 monthly datapoints.<br>b) Post-intervention downward monthly trend in risk with 9 monthly datapoints.<br>Modest reduction in risk of superficial SSIs across all levels of wound contamination pre-compared with post-intervention:<br>1) clean/clean contaminated surgery.<br>2) contaminated/dirty surgery.	% Operations with correct antibiotic prophylaxis	a) −0.5% (95%CI −2.5;1.4)<br>b) −0.7% (95%CI −1.2; −0.1) p 0.027<br>1) RR 0.66 (95%CI 0.49; 0.91) p 0.01<br>2) RR 0.17 (95%CI 0.04; 0.74) p 0.05
Chalker, J. et al. (2002) [58]	1) Agreements with district leaders;<br>2) Workshops to obtain consensus on a) CG from all clinical staff;<br>b) administration staff on duties; c) district supervisors on supervisory methods;<br>3) Workshops on CG and antibiotic dosing for Clinicians 3–4 days.<br>4) Ceremony and donation of money (Committing to responsibilities)	• *N* = 6.270<br>• 6	1-6a) Encounters with an antibiotic: proportion of patients receiving an antibiotic as part of the prescription; 1a) baseline 67% (3870 records) (129 child health services)<br>1-6b) Proportion of patients receiving an adequate dose as per the CG:1b) baseline 30% (2593 prescriptions)<br><br>1) Baseline survey Sept 1994		1a) 67%<br>1b) 30%<br>2a) 46%<br>$P < 0.01$<br>2b) 91% $p < 0.001$<br>3a) 45%<br>3b) 98%<br>$p < 0.01$<br>4a) 48%

	5) Information/education/ communication campaign; 6). Retraining on CGs: diagnoses, treatment, antibiotic doses; 7). Supervision (to check CGs and antibiotic dose followed) 8. Equipment donation 3 times (after 3/6/9 months).		2) survey and 3) supervision: Dec 1995 4) survey and 5) supervision: Sept 1996 6) Final supervision survey: Sept 1997 P-value indicating change based on previous survey/supervision.		4b) 93%<br>5) 43% 5b) 98% $P < 0.01$<br>6a) 40% 6b) 98%
Chandy, S.J. et al. (2014) [37]	Segment 1: developed consensus driven CG. segment 2: disseminated as booklet; segment 3 stable period with no new implementation. segment 4:CG revised and re-distributed. segment 5: CG revised and adapted, disseminated as booklet and electronically.	• *N* = not reported<br>• 110	Overall monthly use (DDD per 100 bed-days) 1) Segment 1 increase in monthly use 0.95 slope (SE 0.18). 2) Segment 2 and 4) antibiotic use exhibited seasonal fluctuations in seasonal trends. 3) Segment 3 showed significant increase in monthly antibiotic use. 5) Segment 5 decline in monthly use. 6) Segment 5 compared with 4: pairwise segmented regression adjusted for seasonality revealed a drop in monthly use equivalent to 10 DDD per 100 BDs (0.401 (SE 0.89).	DDD per 100 bed-days	1) 0.95 (SE 0.18)<br>2) 0.21 (SE 0.08)<br>3) 0.31 (SE 0.06) $p < 0.001$<br>4) 0.05 (SE 0.10)<br>5) −0.37 (SE 0.11) p 0.04<br>6) −0.401 (SE 0.89) p 0.001
Hadi, U. et al. (2008) [43]	Segment 1; new CG developed and endorsed by management; with staff consensus. Segment 2: management declare guideline obligatory; disseminated as booklet; free blood cultures made available. Segment3: interactive teaching sessions and lectures on the guideline, optimal prescribing and AMR. Segment 4: follow-up refresher sessions. Segment 5: post-intervention.	• *N* = 501<br>• 28	DDDs decreased pre-intervention 99.8% to 73% post-intervention.<br><br>Overall, no significant difference in encounters adhering to guideline: 184/212 pre- and 255/289 post-intervention. 1) Baseline: 184/212 2) Post-declaration: 88/103 3) Post-teaching: 104/110 4) Post-refresher course: 63/76 5) Post-intervention: 255/289	DDD per 100 bed-days	1) 87%<br>2) 85%<br>3) 95%<br>4) 83%<br>5) 88%
Wattal, C. et al. (2017) [42]	Introduction of new CG; monthly prescribing audit ; results circulated as feedback to each clinical unit; monthly focus group discussions on	• *N* = 45 (staff only reported)<br>• 15	Baseline: median DDDs per 100 bed-days across all wards: 187.10 DDDs/ 100 bed-days (range 185.88 to 190.67).	DDD per 100 bed-days	1a) −2.5% (190.67; 185.88<br>1b) −1.88% (190.67; 187.14)

	behaviour change strategies that could be implemented to reduce antibiotic consumption.	3 months post intervention: 185.88 DDDs per 100/BDs (−2.5%).<br>6 months post-intervention: 187.14 DDD/100BDs (−1.88%).<br>2) Baseline to 3 months post-intervention antibiotic consumption decreased in 3/35 wards -66.5%, −46.1%, −26.4% DDDs per 100/bed-days respectively (p 0.05).	2)<br>Decrease in 3/35 wards: −66.5%; −46.1%; −26.4%

AMR = antimicrobial resistance; CG = clinical guideline; DDD = defined daily dose; SSIs = surgical site infections.

Table S4. PRISMA 2009 checklist.[71]

Section/Topic	#	Checklist Item	Reported on Page #
		**TITLE**	
Title	1	**Title:** Strategies used for implementing and promoting adherence to antibiotic guidelines in low- and lower-income countries: a systematic review	p1
		**ABSTRACT**	
Structured summary	2	Provide a structured summary including, as applicable: background; objectives; data sources; study eligibility criteria, participants, and interventions; study appraisal and synthesis methods; results; limitations; conclusions and implications of key findings; systematic review registration number.	p1
		**INTRODUCTION**	
Rationale	3	Describe the rationale for the review in the context of what is already known.	1. Introduction p2
Objectives	4	Provide an explicit statement of questions being addressed with reference to participants, interventions, comparisons, outcomes, and study design (PICOS).	PROSPERO (CRD42020153918)
		**METHODS**	
Protocol and registration	5	Indicate if a review protocol exists, if and where it can be accessed (e.g., Web address), and, if available, provide registration information including registration number.	Yes, as above (4)
Eligibility criteria	6	Specify study characteristics (e.g., PICOS, length of follow-up) and report characteristics (e.g., years considered, language, publication status) used as criteria for eligibility, giving rationale.	2.3.1. Inclusion p3
Information sources	7	Describe all information sources (e.g., databases with dates of coverage, contact with study authors to identify additional studies) in the search and date last searched.	*2.2. Search methods* p3
Search	8	Present full electronic search strategy for at least one database, including any limits used, such that it could be repeated.	Sup. file Table S.1.
Study selection	9	State the process for selecting studies (i.e., screening, eligibility, included in systematic review, and, if applicable, included in the meta-analysis).	2.4. *Study quality* p4; Figure 1.
Data collection process	10	Describe method of data extraction from reports (e.g., piloted forms, independently, in duplicate) and any processes for obtaining and confirming data from investigators.	*2.5. Data extraction* p4
Data items	11	List and define all variables for which data were sought (e.g., PICOS, funding sources) and any assumptions and simplifications made.	Table 1. & Sup. file Table 3(a)/(b)
Risk of bias in individual studies	12	Describe methods used for assessing risk of bias of individual studies (including specification of whether this was done at the study or outcome level), and how this information is to be used in any data synthesis.	*2.4. Study quality* p4
Summary measures	13	State the principal summary measures (e.g., risk ratio, difference in means).	*2.6. Data synthesis* p4; Tables 2/3
Synthesis of results	14	Describe the methods of handling data and combining results of studies, if done, including measures of consistency (e.g., I^2) for each meta-analysis.	As above (13)

**Section/Topic**	#	**Checklist Item**	**Reported on Page #**
Risk of bias across studies	15	Specify any assessment of risk of bias that may affect the cumulative evidence (e.g., publication bias, selective reporting within studies).	Sup. file Table S2(2)/(b)
Additional analyses	16	Describe methods of additional analyses (e.g., sensitivity or subgroup analyses, meta-regression), if done, indicating which were pre-specified.	Sup. file Figure. S1.
		**RESULTS**	
Study selection	17	Give numbers of studies screened, assessed for eligibility, and included in the review, with reasons for exclusions at each stage, ideally with a flow diagram.	Figure 1. PRISMA flow chart pp 5/6
Study characteristics	18	For each study, present characteristics for which data were extracted (e.g., study size, PICOS, follow-up period) and provide the citations.	Results pp.5-18; Table 1 & Table 2
Risk of bias within studies	19	Present data on risk of bias of each study and, if available, any outcome level assessment (see item 12).	Sup. file Table S2(a)/(b)
Results of individual studies	20	For all outcomes considered (benefits or harms), present, for each study: (a) simple summary data for each intervention group (b) effect estimates and confidence intervals, ideally with a forest plot.	Results pp 6-13; Table 2; Table 3; Sup. file Table S3(a)/(b)
Synthesis of results	21	Present results of each meta-analysis done, including confidence intervals and measures of consistency.	Table 3
Risk of bias across studies	22	Present results of any assessment of risk of bias across studies (see Item 15).	Sup. file Table S2(a)/(b)
Additional analysis	23	Give results of additional analyses, if done (e.g., sensitivity or subgroup analyses, meta-regression [see Item 16]).	Sup. file Figure. S1.
		**DISCUSSION**	
Summary of evidence	24	Summarise the main findings including the strength of evidence for each main outcome; consider their relevance to key groups (e.g., healthcare providers, users, and policy makers).	Discussion pp18 to 20 Tables 2/ 3
Limitations	25	Discuss limitations at study and outcome level (e.g., risk of bias), and at review-level (e.g., incomplete retrieval of identified research, reporting bias).	Discussion p 19
Conclusions	26	Provide a general interpretation of the results in the context of other evidence, and implications for future research.	Conclusion p 20
		**FUNDING**	
Funding	27	Describe sources of funding for the systematic review and other support (e.g., supply of data); role of funders for the systematic review.	NIL

# = Checklist item number.
